# α9 Nicotinic Acetylcholine Receptor Promotes Tumor Proliferation and Suppresses Ferroptosis in Triple-Negative Breast Cancer

**DOI:** 10.3390/biom15060835

**Published:** 2025-06-08

**Authors:** Xiaoli Feng, Yuxi Tian, Xijun Guo, Josh Haipeng Lei, Jiaqi Yu, Chenglong Zheng, Mingyue Chen, Ren-Bo Ding, Hang Fai Kwok, Sulan Luo, Jiaolin Bao

**Affiliations:** 1 Key Laboratory of Tropical Biological Resources of Ministry of Education, School of Pharmaceutical Sciences, School of Life and Health Sciences, Collaborative Innovation Center of One Health, Hainan University, Haikou 570228, China; 22220860000095@hainanu.edu.cn (X.F.); 22211007000021@hainanu.edu.cn (Y.T.); guoxu@hainanu.edu.cn (X.G.); 23211007000023@hainanu.edu.cn (J.Y.); 23110710000017@hainanu.edu.cn (C.Z.); dingrenbo@hainanu.edu.cn (R.-B.D.); 2 MoE Frontier Science Centre for Precision Oncology, Faculty of Health Sciences, University of Macau, Macau 999078, China; haipenglei@um.edu.mo; 3 National Key Laboratory of Veterinary Public Health and Safety, College of Veterinary Medicine, China Agricultural University, Beijing 100193, China; mychenlj@163.com; 4 Guangxi Key Laboratory of Special Biomedicine, School of Medicine, Guangxi University, Nanning 530004, China; 5 State Key Laboratory of Quality Research in Chinese Medicine, Institute of Chinese Medical Sciences, University of Macau, Macao 999078, China

**Keywords:** nicotinic acetylcholine receptor, α9 nAChR, CHRNA9, triple-negative breast cancer, ferroptosis, proliferation

## Abstract

Breast cancer is a major global health burden with the highest incidence in women, and triple-negative breast cancer (TNBC) stands out as the most malignant subtype. Effective therapeutic targets are urgently needed to develop new therapies for TNBC. Nicotinic acetylcholine receptor is a ligand-gated ion channel receptor that is associated with the advancement of multiple cancers. Notably, α9 nicotinic acetylcholine receptor (α9 nAChR) is less investigated towards its role in cancer. This study sought to clarify the significance of α9 nAChR in TNBC. Firstly, our results uncovered that the expression of CHRNA9 was notably elevated in TNBC tissues and was associated with poor prognosis of TNBC patients. Further, our data indicated that overexpression of α9 nAChR facilitated the growth of TNBC cells, via mechanisms of simultaneously activating AKT-, ERK- and STAT3-mediated proliferation and negatively regulating ferroptosis through promoting SLC7A11/GSH/GPX4 and Keap1/Nrf2/HO1 signaling. Conversely, CHRNA9 knockdown would completely reverse all this signaling, ultimately inhibiting TNBC tumor growth both in vitro and in vivo. Finally, we reported a specific polypeptide antagonist of α9 nAChR, GeXIVA[1,2] and exerted good anti-tumor effects in tumor-bearing mice of TNBC, which indicated a great potential of GeXIVA[1,2] to be further studied as a novel targeted therapy for TNBC. This study provides a scientific basis for establishing α9 nAChR as a novel therapeutic target for TNBC, which is worthy of further development in the future.

## 1. Introduction

Breast cancer constitutes a major global health burden, with the World Health Organization (WHO) reporting 2.3 million new cases and 670,000 deaths annually, making it the fourth leading cause of cancer mortality worldwide [1]. Molecular classification delineates four principal subtypes, each characterized by distinct receptor profiles [2]. The first subtype, Luminal A, is positive for estrogen receptors (ERs) and progesterone receptors (PRs), while negative for human epidermal growth factor receptor 2 (HER2). The second subtype, Luminal B, also expresses ER, but can exhibit a variable expression of PR, and may be either positive or negative for HER2. The third category, HER2 enriched, is defined by the absence of ER and PR, alongside a positive HER2 status. Lastly, triple-negative breast cancer (TNBC) is characterized by the lack of all three markers: ERs, PRs and HER2. And this classification is primarily based on immunohistochemical profiling. TNBC, representing 15–20% of breast malignancies, demonstrates distinct clinicopathological features, including enhanced metastatic potential and poorer prognosis [3,4]. Therapeutically, systemic chemotherapy is the primary adjuvant treatment option due to the absence of ER, PR and HER2 expression in TNBC, rendering it refractory to endocrine therapies and HER2-targeted agents [5]. The urgent requirement for new therapeutic targets and precision medicine strategies to enhance survival outcomes in this aggressive subtype is underscored by these clinical challenges.

Ferroptosis is a unique form of cell death that is characterized by its dependence on the overload of intracellular iron levels. This process is triggered by an imbalance between the generation of reactive oxidants and the presence of antioxidants, which are crucial for maintaining cellular homeostasis. When this balance is disrupted, it results in the excessive accumulation of oxidants, leading to a phenomenon known as lipid peroxidation. This process ultimately causes significant damage to cellular membranes, contributing to the initiation of ferroptotic cell death [6]. Ferroptosis is markedly distinct from other forms of programmed cell death, such as apoptosis, autophagy, pyroptosis and necroptosis, including its morphological, biochemical characteristics and genetic factors. Cells that experience ferroptosis display distinct morphological characteristics that set them apart from those undergoing apoptosis. Unlike apoptosis, which commonly shows features such as chromatin condensation and the formation of apoptotic bodies, ferroptosis does not exhibit these hallmark signs. Instead, ferroptosis demonstrates a unique set of changes, including the shrinkage of mitochondria and a notable reduction in the number of mitochondrial cristae. Additionally, these cells show membrane bubbling, which further highlights the differences in the morphological outcomes of ferroptosis compared to apoptosis [7,8]. Ferroptosis is primarily characterized by the buildup of ferrous ions, a compromised antioxidant system, an elevation in free radicals, a reduction in reduced glutathione (GSH) levels and an increase in lipid peroxidation [9]. The role of ferroptosis is crucial in the occurrence and progression of various diseases, especially in oncology, leading to the increased research interest in this area. There are studies indicated that tumor cells resistant to chemotherapy are more susceptible to ferroptosis. Recent advancements in understanding the molecular mechanisms of ferroptosis have yielded new exploitable targets for cancer treatment. Multiple signaling pathways have been identified to influence the ferroptosis process, including the SLC7A11/GPX4 pathway, the iron ion metabolism pathway, the lipid metabolism pathway, the p53/STA1/ALOX15 pathway and the p62/Keap1/Nrf2 pathway [10,11,12,13,14].

Numerous diseases can be addressed through the application of ferroptosis inhibitors or inducers. For instance, increasing intracellular Fe(II) levels using heme and ferric chloride can effectively trigger ferroptosis. Conversely, iron chelators such as deferoxamine and lipophilic antioxidants inhibit ferroptosis by mitigating oxidative damage [15,16]. Research has demonstrated that ferroptosis possesses tumor-suppressive properties, thus presenting a potential therapeutic approach for cancer through the promotion of ferroptosis. Existing literature emphasizes the critical importance of ferroptosis in breast cancer treatment. Inducers of ferroptosis, including RSL3 and erastin, can initiate ferroptosis, resulting in breast cancer cell death [17]. Additionally, several clinically utilized drugs, including Sorafenib, Sulfasalazine, and statins, have demonstrated the ability to trigger ferroptosis in tumor cells, thereby amplifying the anti-cancer effect [18]. Therefore, modulating the occurrence and progression of ferroptosis in tumor cells may offer novel therapeutic strategies for TNBC patients [19].

Nicotinic acetylcholine receptors (nAChRs) are ligand-gated ion channel receptors, which are widely expressed not only in the central and peripheral nervous system, but also in non-neuronal cells such as muscle, skin, pancreas and lung. According to the expression site, nAChRs can be divided into neuronal type and muscle type [20]. Previous evidence demonstrated that silencing the expression of α9 nAChR (α9 nicotinic acetylcholine receptor, encoded by CHRNA9 gene, in cancer cells resulted in a significant reduction in the levels of metastasis-related proteins, Slug and Vimentin [21]. This observation indicates that α9 nAChR could be essential in the metastatic process of tumor cells. Furthermore, it has been noted that the expression of tumor suppressors p21 and p27 markedly increases under conditions of low α9 nAChR expression [21]. The ER and activator protein-1 (AP1) can effectively bind to the α9 nAChR promoter, a process that is essential for the regulation of α9 nAChR expression. Research also indicates that treatment with nicotine and estrogen (E2) significantly upregulates the expression of α9 nAChR [22]. Additionally, by activating α9 nAChR expression, nicotine substantially enhances the expression of mannose-binding protein 3 (LGALS3) in cancer cells [23], thereby improving their anti-apoptotic capabilities and migratory potential. In experiments using the normal human mammary epithelial cells HBL-100, exposure to secondhand smoke significantly elevated α9 nAChR expression level in these cells [24]. Concurrently, treatment with secondhand smoke also promoted the expression of focal adhesion kinase (FAK), a crucial marker associated with cell migration [24]. Therefore, we conclude that α9 nAChR plays a significant role in the processes associated with both the development and progression of cancer. It is noteworthy that αO-conotoxin GeXIVA, a novel αO-superfamily conotoxin first discovered by our research group, is derived from the marine species *Conus generalis* and composes of a chain of 28 amino acid residues [25]. It acts as a specific antagonist for the α9 nAChR [25].

However, research on the role of α9 nAChR in the progression of TNBC, along with its underlying molecular mechanisms, remains limited. To address this research gap, the present study sought to explore the oncogenic role of α9 nAChR in TNBC and its molecular mechanisms, thereby providing scientific evidence and theoretical guidance for considering α9 nAChR as a therapeutic target for this type of cancer. Previous research has indicated that ferroptosis suppression is a critical factor that facilitates the progression of cancer [26,27]. And ferroptosis induction plays an important role in cancer treatment [6,28]. Thus, the induction of ferroptosis has emerged as a vital mechanism in cancer treatment, suggesting that promoting this form of cell death could be leveraged as a therapeutic strategy, including breast cancer. However, the specific effect of α9 nAChR on ferroptosis in TNBC is unclear [29].

In this study, we firstly analyzed public databases to identify the relationship between α9 nAChR expression and the prognosis of TNBC patients. We further assessed the impact of α9 nAChR overexpression or knockdown in TNBC cell lines, including HCC38 and HCC1937, on cell proliferation-, apoptosis- and ferroptosis-associated signaling pathways. Finally, we also examined the in vivo antitumor effect of α9 nAChR blockage via shRNA and antagonist in TNBC mouse models.

## 2. Materials and Methods

### 2.1. Cell Lines and Cell Culture

Human TNBC cell lines HCC38 and HCC1937 were purchased from the Procell Life Science and Technology Co., Ltd. (Wuhan, China). All cell lines used in this study were confirmed to be free from mycoplasma contamination. Cells were cultured in either RPMI 1640 (PM150110, Procell) or DMEM (PM150210, Procell) medium, both of which were supplemented with 10% fetal bovine serum (FBS) (164210, Procell) and 1% Penicillin/Streptomycin (PB180120, Procell) in a humidified incubator containing 5% CO_2_ at a temperature of 37 °C.

### 2.2. Colony Formation Assay

Cell proliferation ability was measured by plate colony formation assay. Briefly, 1000 HCC38 cells or 500 HCC1937 cells were added to each well of a six-well plate and incubated for 2 to 3 weeks until colonies were obviously formed; the medium was regularly changed. In this experiment, the overexpression groups (CHRNA9 and Ctrl) was cultured for 15 days and the knockdown groups (shCHRNA9-1, shCHRNA9-2 and shCtrl) was cultured for 23 days to terminate the experiment. Subsequently, the plate was carefully cleaned and treated with crystal violet and the number of colonies was counted.

### 2.3. Measurement of Cell Viability

The CellTiter-Lumi™ Steady II Cell Viability Assay Kit (C0058XL, Beyotime, Shanghai, China) was used to quantify ATP content. Since ATP content can well reflect the number of living cells and ATP content is proportional to the chemiluminescence intensity, it can be simply calculated from the chemiluminescence intensity of cell viability or cell number. Briefly, different treatments of cells were cultured for 0 to 96 h. Following the guidelines set forth by the manufacturer, the cells were treated with the supplied reaction mixture for a duration of 10 min at ambient temperature. A microtiter plate reader equipped with a chemiluminescence module was used to measure the absorbance.

### 2.4. Plasmid Constructions and Cell Transfection Protocol

The CHRNA9 CDS sequence was subcloned into the pCDH-GFP vector to overexpress CHRNA9 gene and CHRNA9 overexpression vector was purchased from Sangon Biotech (Shanghai, China). CHRNA9 shRNA sequences were subcloned into the pLKO.1 vector. The shRNA sequence of CHRNA9 knockdown is shown below: hCHRNA9 shRNA F-1: CCGGGGGTGACTGGCCTCTAGTTTACTCGAGTAAACTAGAGGCCAGTCACCCTTTTTG; hCHRNA9 shRNA R-1: AATTCAAAAAGGGTGACTGGCCTCTAGTTTACTCGAGTAAACTAGAGGCCAGTCACCC; hCHRNA9 shRNA F-2: CCGGCCTGATAGGTAAATACTACATCTCGAGATGTAGTATTTACCTATCAGGTTTTTG; hCHRNA9 shRNA R-2: AATTCAAAAACCTGATAGGTAAATACTACATCTCGAGATGTAGTATTTACCTATCAGG. We transfected the constructed CHRNA9 knockdown/overexpression plasmid with VSVG and pCMV delta R8.28 into HEK293T for a packing lentivirus. The lentivirus was used to infect HCC38 and HCC1937 cell line with medium containing 8 μg/mL polybrene. The cells were incubated at 37 °C for 24 h and then replaced by a suitable medium with 10% FBS. The stable expression cell lines could be selected with suitable puromycin. Verification of these stable expression cell lines was conducted through Western blot. The plasmid transfected in the Ctrl group is the empty vector pCDH-GFP of the overexpression plasmid, serving as the control for the overexpression group; whereas the plasmid transfected in the shCtrl group is the empty vector pLKO.1, serving as the control for shCHRNA9-1/2.

### 2.5. Determination of Intracellular ROS, Malondialdehyde (MDA) and Glutathione (GSH)

The levels of intracellular reactive oxygen species (ROS) were measured using flow cytometry in conjunction with the Reactive Oxygen Species Assay Kit (Beyotime, S0033S), following the provided protocol. In summary, cells were treated with 10 μM DCFH-DA following a 48-h exposure to erastin. After 20 min of DCFH-DA incubation, the cells were harvested for the measurement of fluorescence intensity. To assess the production of malondialdehyde (MDA) and reduced glutathione (GSH) in breast cancer cells, the Cell Malondialdehyde (MDA) assay kit (Colorimetric method) and Reduced glutathione (GSH) assay kit (Nanjing Jiancheng Bioengineering Institute, Nanjing, China) were used.

### 2.6. Western Blot (WB)

Cell lysates were prepared using a radioimmunoprecipitation assay (RIPA) buffer, which was supplemented with a proteinase inhibitor to ensure the preservation of protein integrity. To quantify the protein concentrations in the lysates, we employed the BCA Protein Assay Kit (Beyotime, Shanghai, China). For the subsequent analysis, 20 µg of the extracted proteins were subjected to separation by sodium dodecyl sulfate-polyacrylamide gel electrophoresis (SDS-PAGE). Following this separation, the proteins were electroblotted onto polyvinylidene difluoride (PVDF) membranes (SEQ00010, Merck Millipore, Darmstadt, Germany). In preparation for the antibody incubation, the membranes underwent a blocking step in TBST containing 3% bovine serum albumin (BSA) for 1 h and then incubated with the following appropriate primary antibodies: BAX (1:2000, 50599-2-Ig, Proteintech, Wuhan, China), Bcl-2 (1:1000, AF6139, Affinity Biosciences, Changzhou, China), GPX4 (1:1000, 67763-1-Ig, Proteintech), p-AKT (Ser473) (1:1500, 66444-1-Ig, Proteintech), AKT (1:2000, 60203-2-Ig, Proteintech), p-ERK (Thr202/Tyr204) (1:1000, AF1015, Affinity Biosciences), ERK (1:1000, BF8004, Affinity Biosciences), GAPDH (1:10,000, 10494-1-AP, Proteintech), p-STAT3 (Tyr705) (1:1000, AF3293, Affinity Biosciences), STAT3(1:1000, AF6294, Affinity Biosciences), SLC7A11 (1:1000; DF12509, Affinity Biosciences), CHRNA9 (1:5000; PA5-46826, Thermo Fisher), α-tubulin (1:5000; 66031-1-lg; Proteintech), Keap1 (1:5000; 10503-2-AP, Proteintech), Nrf2 (1:1000; AF0639; Affinity Biosciences), HO-1 (1:1000; AF5393; Affinity Biosciences), PD-L1 (1:4000; 66248-1-Ig; Proteintech), and IDO1 (1:1000; 13268-1-AP; Proteintech) at 4 °C overnight. Following three washes with TBST (each lasting 10 min), the membranes were treated with secondary antibodies that had been diluted in TBST for 1 h at room temperature. Subsequently, the samples underwent an additional three washes with TBST, after which enhanced chemiluminescence (ECL) was conducted using an ECL kit (WBKLS0500, Merck Millipore).

### 2.7. In Vivo Animal Study

Female BALB/c nude mice aged 4 to 6 weeks and 6-week-old female BALB/C mice were obtained from Guangzhou Yancheng Biotechnology Company Limited (Guangzhou, China). Mice were kept in a controlled environment featuring a temperature of 24 °C and relative humidity of 50–60%, under a 12-h light/dark cycle and had unrestricted access to standard chow and water. Following a 3-day acclimatization period for both the BALB/c nude and BALB/c female mice, they were inoculated subcutaneously with breast cancer cells to establish tumor models.

In the HCC38 xenograft model, a suspension of HCC38 cells (4 × 10^6^), which were stably transfected with either shCHRNA9 or a control shRNA (shCtrl), was introduced subcutaneously into the flanks of BALB/c nude mice (*n* = 6). Specifically, the cells were resuspended in 100 μL of phosphate-buffered saline (PBS) and implanted into the right flank region of each mouse. To monitor tumor development, the size of the tumors was measured using calipers at 2-week intervals.

In the 4T1 allograft mouse model, a total of 4T1 cells (5 × 10^5^) were injected subcutaneously into each BALB/c mouse (*n* = 6). Following the initial tumor cell inoculation, treatment commenced on the eighth day with GeXIVA administered at a dosage of 17 μg/kg. This treatment was given through subcutaneous injection once daily in close proximity to the tumor site. Tumor growth was closely monitored, with measurements taken every other day beginning on the seventh day post-inoculation.

The volume of the tumors was calculated utilizing the following formula: volume = length × (width)^2^/2. At the conclusion of the experiment, the mice were euthanized to allow for the collection of data; the subcutaneous tumors were then excised, weighed and photographed for further analysis.

### 2.8. Transcriptome and Clincial Data Information

The transcriptome data of 125 TNBC tissues and 112 adjacent normal tissues were downloaded from The Cancer Genome Atlas (TCGA) database. RNA reads were normalized using the edgeR package and the Trimmed mean of M-value (TMM) was applied as a gene expression value. The expression difference between TNBC and normal groups was visualized by the ggpubr package. For the Kaplan–Meier analysis, paired TNBC transcriptome data and clinical follow-up information from the METABRIC dataset was download from the cBioPortal database. In total, 250 TNBC patients were with complete data of CHRNA9 expression value and overall survival time (did not die of other causes). CHRNA9 high expression was defined by Z-Socre value > 0 and CHRNA9 low expression was defined by Z-Socre value < 0. The Kaplan–Meier analysis was conducted with GraphPad Prism software(ver.8.3.0 for Windows, GraphPad Software, Inc., San Diego, CA, USA).

### 2.9. Statistical Analysis

Statistical analyses were conducted using GraphPad Prism. Comparisons between groups were executed through one-way ANOVA, two-way ANOVA and *t*-test. The statistical significance of the Kaplan–Meier analysis was assessed by Log-rank (Mantel-Cox) test. All data with *p* < 0.05 were defined statistically significant (* *p* < 0.05; ** *p* < 0.01; *** *p* < 0.001; ns, not significant (*p* > 0.05).

## 3. Results

### 3.1. High CHRNA9 Expression Is Associated with Poor Prognosis in TNBC Patients

**Figure 1:**
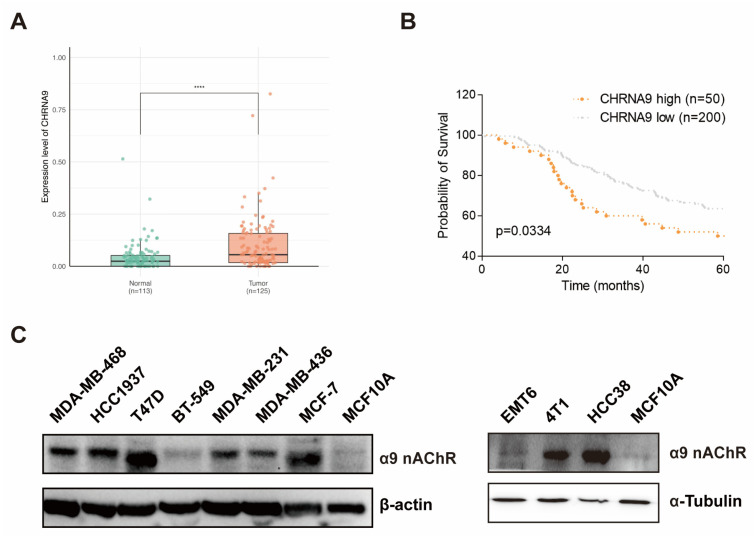
High CHRNA9 expression is associated with poor prognosis in TNBC patients. (**A**) The expression of CHRNA9 was elevated in TNBC tissues (*n* = 125) compared to adjacent normal tissues (*n* = 113). Data accessed from The Cancer Genome Atlas (TCGA) database. **** *p* < 0.0001. (**B**) TNBC patients with high CHRNA9 expression (*n* = 50) exhibited a worse survival time than those with low CHRNA9 expression (*n* = 200). Data accessed from the METABRIC dataset in the cBioPortal database. The statistical significance was assessed by Log-rank (Mantel–Cox) test. The *p*-value is indicated, as shown in the diagram. (**C**) α9 nAChR protein expressions in breast cancer cell lines (TNBC: MDA-MB-468, HCC1937, BT549, MDA-MB-231, MDA-MB-469, EMT6, 4T1 and HCC38; non-TNBC: T47D and MCF-7) and the normal breast epithelial cell line (MCF10A). (original western blot images see Supplementary Materials)

At the beginning, we wanted to know the expression pattern of CHRNA9 between TNBC tumors and normal tissues and whether CHRNA9 has a potential impact on the prognosis of TNBC patients. We used transcriptional data from a cohort of breast cancer patients consisting of 125 TNBC tumors and 112 adjacent normal tissues from The Cancer Genome Atlas (TCGA) database. We compared the mRNA expression levels of CHRNA9 in these two groups and found that CHRNA9 exhibited significantly higher expression in TNBC tumors than in normal tissues (Figure 1A). Moreover, we also downloaded data of a breast cancer cohort with paired tumor transcriptome data and clinical follow up information from the METABRIC dataset in the cBioPortal database. The Kaplan–Meier analysis revealed that CHRNA9 high-expression TNBC patients (*n* = 50) exhibited significantly worse prognosis than CHRNA9 low-expression TNBC group (*n* = 200) (Figure 1B). We also examined the α9 nAChR protein expression levels in 10 breast cancer cell lines and compared their expressions with normal breast epithelial cell line MCF10A by Western blot analysis, the results demonstrated that α9 nAChR protein generally exhibited higher expression levels in the majority of breast cancer cell lines (including TNBC cell lines MDA-MB-468, HCC1937, MDA-MB-231, MDA-MB-436, 4T1 and HCC38) than in normal MCF10A cells (Figure 1C). Collectively, these findings indicated that CHRNA9/α9 nAChR expression was elevated in TNBC tissues and was associated with poor prognosis of TNBC patients, suggesting that CHRNA9/α9 nAChR may play an important role in the occurrence and development of TNBC.

### 3.2. α9 nAChR Promoted TNBC Cell Growth In Vitro

**Figure 2:**
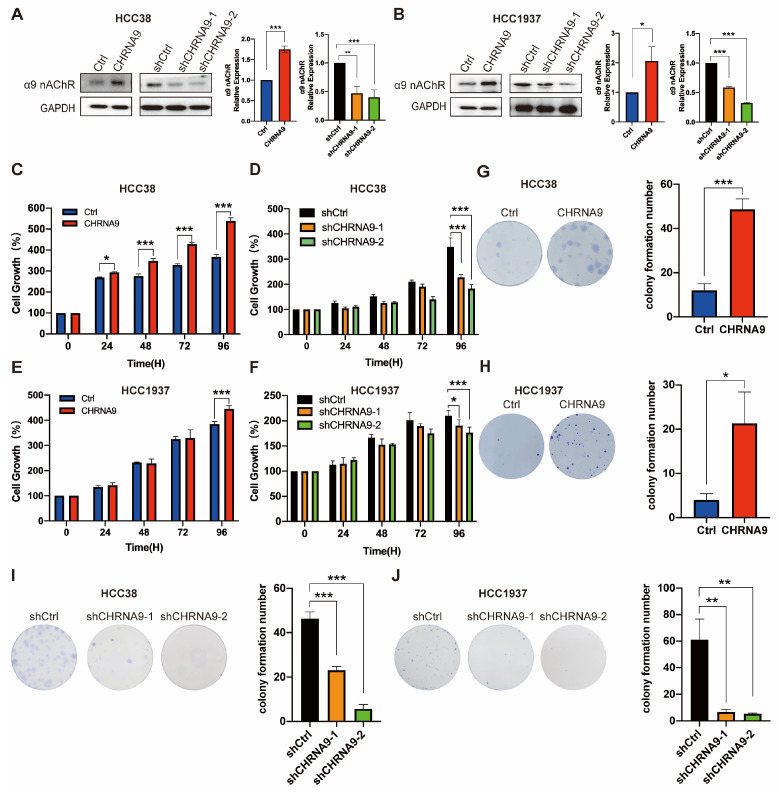
α9 nAChR facilitated TNBC cell growth in vitro. (**A**,**B**) Western blot results demonstrating the overexpression and knockdown efficiencies of α9 nAChR. (original western blot images see Supplementary Materials) Graphs are representatives of three independent experiments. (**C**,**E**) CHRNA9 overexpression promoted cell growth in HCC38 and HCC1937 cells examined by ATP assay. *n* = 3 biological replicates. (**D**,**F**) CHRNA9 knockdown inhibited cell growth in HCC38 and HCC1937 cells examined by ATP assay. *n* = 3 biological replicates. (**G**,**H**) The colony formation experiment demonstrated CHRNA9 overexpression promoted cell growth in HCC38 and HCC1937 cells. The experiment was conducted for 15 days. *n* = 3 biological replicates. (**I**,**J**) The colony formation experiment demonstrated CHRNA9 knockdown inhibited cell growth in HCC38 and HCC1937 cells. The experiment was conducted for 23 days. *n* = 3 biological replicates. * *p* < 0.05; ** *p* < 0.01; *** *p* < 0.001.

To investigate whether α9 nAChR affects TNBC cell growth, we overexpressed and knockdown CHRNA9 gene in HCC38 and HCC1937 cells by lentiviruses. Western blot was used to verify the overexpression and knockdown efficiencies of α9 nAChR (Figure 2A,B). We next investigated the effect of α9 nAChR on growth of TNBC cells by ATP assay and colony formation. The results of ATP assay are shown in Figure 2C–F. Significant differences in cell growth rates were observed between the control group and the CHRNA9 overexpression group in both HCC38 and HCC1937 cells (Figure 2C,E). Conversely, after knockdown of CHRNA9, the cell survival rates of both HCC38 and HCC1937 cells showed a decreasing trend, with significant differences observed at 96 h (Figure 2D,F). Consistent findings were also demonstrated with a colony formation experiment. As shown in Figure 2I,J, CHRNA9 knockdown induced a significant reduction in the number of colonies, while overexpressing CHRNA9 remarkably promoted the clonogenic growth of HCC38 and HCC1937 cells. Collectively, these results demonstrated that overexpression of α9 nAChR promoted the growth of TNBC cells, while knockdown of α9 nAChR inhibited TNBC cell growth.

### 3.3. α9 nAChR-Regulated TNBC Cell Proliferation Was Associated with AKT-, ERK- and STAT3-Mediated Signaling Pathways

**Figure 3:**
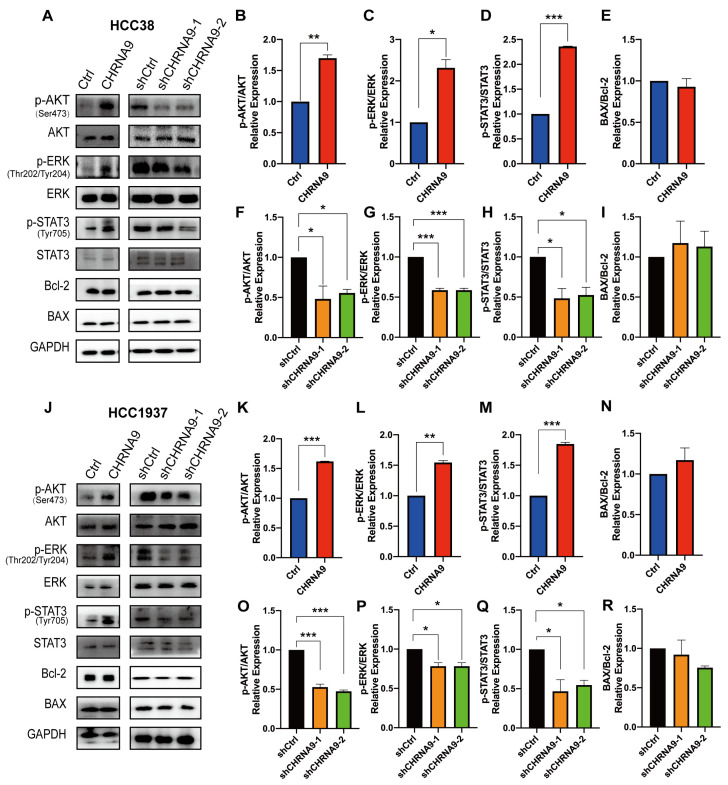
α9 nAChR regulated the AKT, ERK and STAT3 signaling pathways. (**A**) Western blot analysis on proliferation and apoptosis pathway-related protein changes in HCC38 cells. The densitometric analysis of phospho-AKT (Ser473)/AKT ratio (**B**,**F**), phospho-ERK (Thr202/Tyr204) /ERK ratio (**C**,**G**), phospho-STAT3 (Tyr705)/STAT3 ratio (**D**,**H**) and BAX/Bcl-2 ratio (**E**,**I**) in HCC38 cells (**J**) Western blot analysis on proliferation and apoptosis pathway-related protein changes in HCC1937 cells. The densitometric analysis of phospho-AKT (Ser473)/AKT ratio (**K**,**O**), phospho-ERK (Thr202/Tyr204)/ERK ratio (**L**,**P**), phospho-STAT3 (Tyr705)/STAT3 ratio (**M**,**Q**) and BAX/Bcl-2 ratio (**N**,**R**) in HCC1937 cells. Graphs (**A**,**J**) are representatives of three independent experiments. * *p* < 0.05; ** *p* < 0.01; *** *p* < 0.001. (original western blot images see Supplementary Materials)

To gain further understanding on the underlying mechanisms of how α9 nAChR promotes TNBC cell growth, we first investigated the upstream signaling pathways responsible for cancer cell proliferation. PI3K-Akt-mTOR, MAPK/ERK and JAK-STAT3 signaling were three most classical signaling pathways responsible for promoting cellular proliferation. AKT, ERK and STAT3 signaling also play essential roles in regulating the proliferation and survival of cancer cells [30]. Western blot analysis showed that phosphorylation activation of AKT (at Ser473), ERK (at Thr202/Tyr204) and STAT3 (at Tyr705) were significantly upregulated by CHRNA9 overexpression, whereas they were suppressed by CHRNA9 knockdown in both TNBC cell lines (HCC38 and HCC1937) (Figure 3). These results indicated that AKT-, ERK- and STAT3-mediated signaling pathways were involved in the α9 nAChR-induced promotion of TNBC cell proliferation.

### 3.4. α9 nAChR Promoted TNBC Cell Growth by Negatively Regulating Ferroptosis, but Not Apoptosis

In addition to proliferation, cancer cell growth is also highly associated with programmed cell death, and apoptosis and ferroptosis are major players in this process. Apoptosis is a classical type of programmed cell death with the BAX/Bcl-2 ratio frequently utilized as an apoptotic indicator. To determine whether the α9 nAChR-regulated cell proliferation is also associated with the anti-apoptotic pathway, we examined the expressions of apoptosis-related BAX and Bcl2. As illustrated in Figure 3, there was no significant change in the BAX/Bcl-2 ratio upon CHRNA9 manipulation in both TNBC cells, suggesting that α9 nAChR does not influence the proliferation of TNBC cells through anti-apoptotic mechanisms.

**Figure 4:**
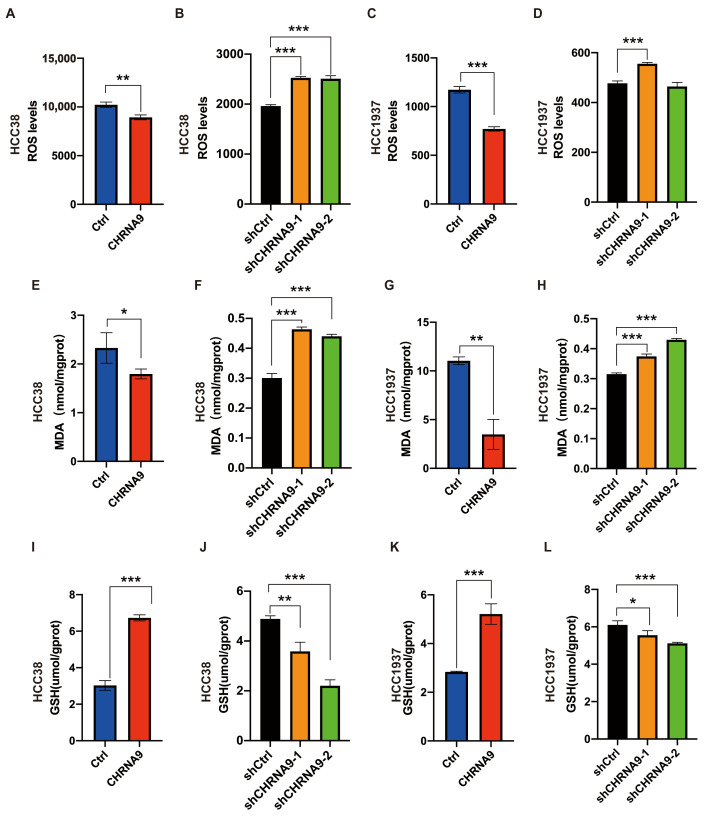
α9 nAChR negatively regulated ferroptosis in TNBC cells. Intracellular ROS (**A**–**D**), MDA (**E**–**H**) and GSH (**I**–**L**) levels were determined in HCC38 and HCC1937 cells upon CHRNA9 manipulation. *n* = 3 biological replicates. For comparing control and CHRNA9 overexpression groups, 25 μM erastin pre-treatment was applied to induce ferroptosis condition in HCC38 and HCC1937 cells. * *p* < 0.05; ** *p* < 0.01; *** *p* < 0.001.

Ferroptosis is an iron-dependent programmed cell death and is characterized by heightened oxidative stress within cells [9]. A key characteristic of oxidative stress is the variation in reactive oxygen species (ROS) levels. An essential process in ferroptosis is lipid peroxidation, with malondialdehyde (MDA) being the most significant byproduct produced during this phenomenon [31]. So, ROS and MDA are established as phenotypic indicators of ferroptosis. To investigate the regulatory effects of α9 nAChR on ferroptosis, we examined the intracellular ROS and MDA levels upon CHRNA9 manipulation in TNBC cells (Figure 4). Under the condition of ferroptosis induction by erastin, overexpression of CHRNA9 significantly attenuated the erastin-elevated ROS and MDA levels, whereas knockdown of CHRNA9 triggered the occurrence of ferroptosis, as evidenced by significant elevated ROS and MDA levels (Figure 4). These findings indicated that α9 nAChR promoted TNBC cell growth by negatively regulating ferroptosis.

### 3.5. α9 nAChR Suppressed Ferroptosis via Promoting SLC7A11/GPX4 and Keap1/Nrf2/HO1 Signaling

**Figure 5:**
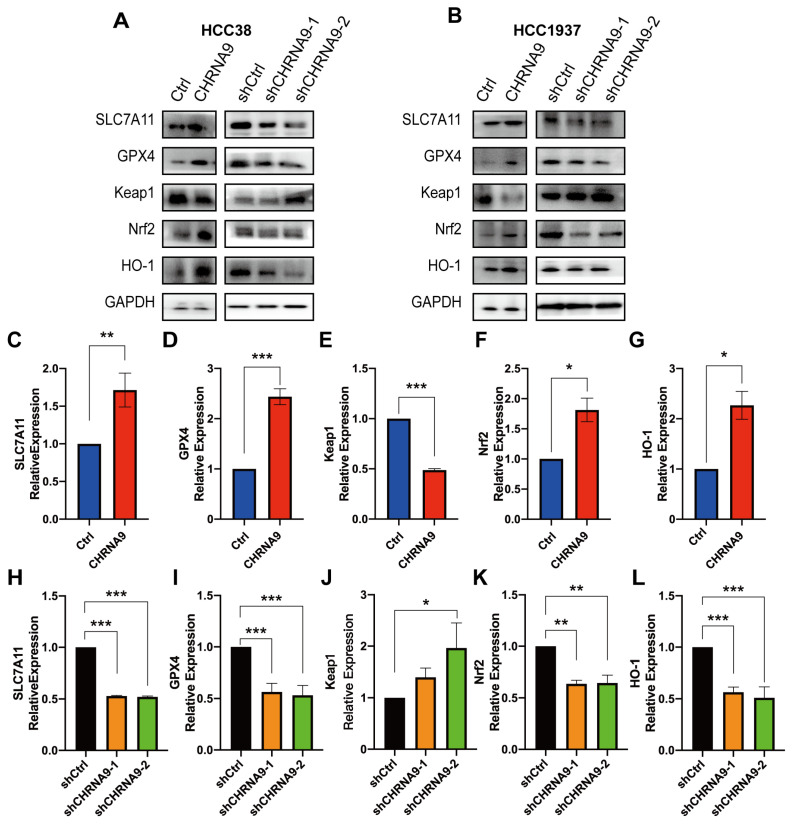
α9 nAChR suppressed ferroptosis via promoting SLC7A11/GPX4 and Keap1/Nrf2/HO1 signaling. (**A**,**B**) Western blot analysis on ferroptosis pathway-related protein changes in HCC38 and HCC1937 cells. Graphs are representatives of three independent experiments. The densitometric analysis of SLC7A11 (**C**,**H**), GPX4 (**D**,**I**), Keap1 (**E**,**J**), Nrf2 (**G**,**K**) and HO-1 (**F**,**L**) in HCC38 cells. For comparing control and CHRNA9 overexpression groups, 25 μM erastin pre-treatment was applied to induce ferroptosis conditions in HCC38 cells. * *p* < 0.05; ** *p* < 0.01; *** *p* < 0.001. (original western blot images see Supplementary Materials)

To further confirm the α9 nAChR-regulated ferroptosis and explore its associated mechanism, we investigated the canonical regulatory pathway of ferroptosis, the SLC7A11/GSH/GPX4 signaling (System Xc-). As shown in Figure 4I–L and Figure 5, overexpression of CHRNA9 significantly upregulated the erastin-depleted SLC7A11 and GPX4 protein expression and GSH levels in TNBC cells, indicating CHRNA9 overexpression was able to reverse erastin-induced ferroptosis via activating SLC7A11/GSH/GPX4 signaling. Consistently, CHRNA9 knockdown-triggered ferroptosis was also associated with significant decline in levels of SLC7A11, GSH and GPX4. In addition to SLC7A11/GSH/GPX4 signaling, the Keap1/Nrf2/HO-1 signaling also plays an important role in regulating ferroptosis. Western blot results showed that overexpression of CHRNA9 significantly downregulated Keap1 expression and upregulated Nrf2 and HO-1 expression in erastin-induced ferroptosis conditions, whereas CHRNA9 knockdown abolished the activation of Keap1/Nrf2/HO-1 signaling in TNBC cells. These findings collectively indicated that α9 nAChR modulated ferroptosis suppression in TNBC mainly through dually promoting SLC7A11/GPX4 and Keap1/Nrf2/HO1 signaling, ultimately maintaining tumor cell survival.

### 3.6. α9 nAChR Blockage Inhibited TNBC Tumor Growth In Vivo

**Figure 6:**
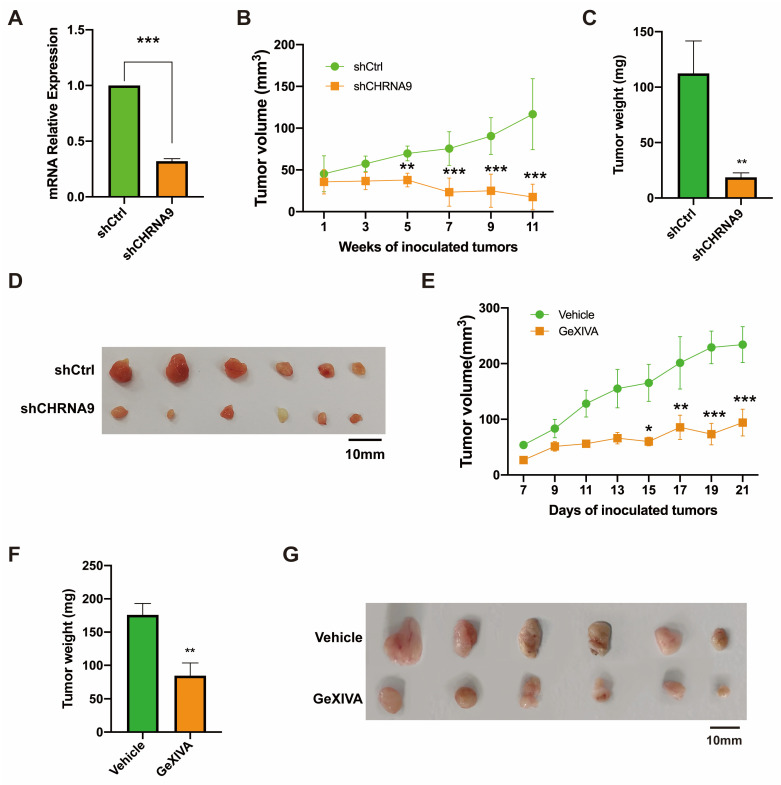
The anti-tumor effect of α9 nAChR blockage by shRNA or antagonist in TNBC. (**A**–**D**) CHRNA9 knockdown inhibited TNBC tumor growth in vivo. (**A**) Validation of the stable CHRNA9 knockdown status in HCC38 xenograft tumors. (**B**) Tumor growth curves of HCC38 xenograft nude mice (*n* = 6). (**C**) Tumor weights were stripped from HCC38 xenograft mice at the endpoint of the experiment (*n* = 6). (**D**) Tumor images of HCC38 nude mice sacrificed at the endpoint of the experiment. (**E**–**G**) The specific α 9 nAChR antagonist GeXIVA[1,2] inhibited TNBC tumor growth in vivo. (**E**) Tumor growth curves of 4T1 allograft mice (*n* = 6). (**F**) Tumor weights were stripped from 4T1 allograft mice at the endpoint of the experiment (*n* = 6). (**G**) Tumor images of 4T1 tumor-bearing mice sacrificed at the endpoint of the experiment. * *p* < 0.05; ** *p* < 0.01; *** *p* < 0.001.

Based on the above in vitro finding, we have reasons to hypothesize that α9 nAChR could be a therapeutic target for TNBC. We first evaluated the in vivo anti-tumor effect of direct silencing CHRNA9 by shRNA in a TNBC xenograft model. HCC38 cells of stable CHRNA9 knockdown or control were injected into the dorsal flank of BALB/c nude mice and the tumor volumes were monitored. As shown in Figure 6A–D, CHRNA9 knockdown induced a significant reduction in tumor volumes and tumor weights compared to the control group, which suggested α9 nAChR should be a useful therapeutic target for TNBC. Previously, our group discovered a specific polypeptide antagonist of α9 nAChR from Conus generalis, named as GeXIVA[1,2] [25]. We therefore applied GeXIVA[1,2] in subcutaneous 4T1 tumor-bearing mice. The result showed that GeXIVA[1,2] treatment exhibited good anti-tumor effects (Figure 6E–G). Altogether, these in vivo results clearly demonstrated α9 nAChR is a therapeutic target for TNBC and GeXIVA[1,2] has a great potential to be further studied as a novel targeted therapy for TNBC.

## 4. Discussion

**Figure 7:**
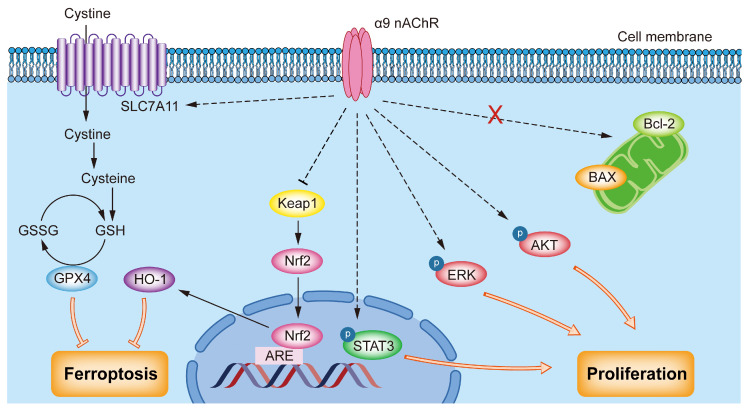
Schematic diagram of the oncogenic mechanism of α9 nAChR in TNBC. α9 nAChR promotes proliferation by activating the AKT, ERK and STAT3 signaling pathways and suppressing ferroptosis via promoting SLC7A11/GSH/GPX4 and Keap1/Nrf2/HO-1 pathways.

α9 nAChR is abnormally highly expressed in malignant tumors such as lung cancer, melanoma and colorectal cancer [32]. Our results revealed that the expression level of α9 nAChR mRNA in the tumors of TNBC patients was significantly higher than that in a normal control group. There was a significant difference in the 5-year survival rate between the CHRNA9 high expression and CHRNA9 low expression groups. TNBC patients with high CHRNA9 expression were associated with significantly worse prognosis. These results indicated that α9 nAChR may play an important role in the occurrence and development of TNBC. It is also worthwhile to further study the possibility of α9 nAChR as a potential therapeutic target in order to provide new treatment strategy for TNBC. Therefore, based on the above findings, we continued to explore the oncogenic mechanism of α9 nAChR in regulating tumor growth of TNBC. Our study demonstrated that overexpression of CHRNA9 significantly promoted the growth of TNBC cells, while knockdown of CHRNA9 markedly suppressed TNBC cell growth. The underlying mechanism uncovered by the present study demonstrated that α9 nAChR was able to activate AKT-, ERK- and STAT3-mediated signaling pathways and suppressed ferroptosis via promoting SLC7A11/GSH/GPX4 and Keap1/Nrf2/HO1 signaling, ultimately supporting the continuous tumor cell growth of TNBC (Figure 7).

Currently, a considerable number of studies have explored the role of α7 nAChR in tumorigenesis and development. For example, in non-small cell lung cancer cells, α7 nAChR regulated cell proliferation by activating the AKT and ERK signaling pathways [33]. Furthermore, α7 nAChR inhibited apoptosis by activating the JAK2-NF-κB and JAK2-STAT3 pathways, thereby promoting the expression of the anti-apoptotic protein Bcl-2 [34]. Additionally, nicotine exerted anti-apoptotic effects in colorectal cancer cells by activating the AKT and ERK signaling pathways through α7 nAChR [35]. Thus, α7 nAChR can play regulatory roles in cancer through various pathways. However, the molecular mechanisms of α9 nAChR in regulating tumorigenesis and development have not been thoroughly investigated. Therefore, exploring the molecular mechanisms of α9 nAChR in promoting cancer growth is essential, especially in some difficult-to-treat cancer types such as TNBC.

AKT is a serine/threonine protein kinase that has been extensively studied for its involvement in tumor development. For instance, reduced levels of AKT have been shown to inhibit the proliferation of cancer cells [36,37]. The STAT3 protein is critical for various cellular processes, including proliferation, differentiation, survival, inflammatory responses, immune processes and angiogenesis [37]. Notably, STAT3 exhibits abnormal activation in several types of cancer cells, such as those found in breast cancer, prostate cancer, head and neck cancer, colon cancer, lung cancer and multiple myeloma [38]. Continuous activation of STAT3 promotes cell cycle progression and enhances the invasion, metastatic potential and angiogenesis of tumors [39,40]. Given the roles of AKT and STAT3 in tumor proliferation, we assessed the expression levels of AKT, STAT3 and their phosphorylated activation forms using Western blot analysis. Our findings demonstrated that overexpression of α9 nAChR enhanced the phosphorylation activation of AKT and STAT3 in TNBC cells.

The mitogen-activated protein kinase (MAPK) signaling pathway includes key components such as RAS, RAF, MEK and ERK, and represents a highly conserved signal transduction cascade throughout evolution [41]. This pathway is crucial to regulating essential cellular processes, including cell survival and proliferation. Upon receiving stimulation from receptor kinases on the cell surface, signals are relayed to MAPK/ERK kinase MEK through a series of cascade reactions. This process enables the simultaneous phosphorylation of ERK1 and ERK2, facilitating physiological processes such as cell proliferation, growth, and migration [42]. The downregulation of ERK signaling has been associated with the inhibition of cancer cell proliferation [43]. Given the role of ERK in promoting cell proliferation, the present study also investigated the effect of α9 nAChR on ERK activation. Our results revealed that α9 nAChR overexpression increased the expression of phosphorylated ERK in TNBC cells.

Ferroptosis represents a novel form of programmed cell death, distinct from apoptosis, autophagy and other forms of cell death [6]. The regulation of ferroptosis is not only involved in tumorigenesis but also provides new therapeutic strategies for cancer treatment. Activation of α7 nAChR has been shown to promote the autophagy and ferroptosis pathways, primarily through increasing the p62 expression, thus further inducing ferroptosis [44]. Currently, there is limited research on the role of α9 nAChR in the relationship with ferroptosis. Our experimental results demonstrated that overexpression of α9 nAChR protected tumor cells from erastin-induced ferroptosis and CHRNA9 knockdown would trigger the occurrence of ferroptosis in cancer cells. Ferroptosis is primarily regulated by the canonical SLC7A11/GSH/GPX4 and antioxidant Keap1/Nrf2/HO-1 signaling pathways. Specifically, α9 nAChR upregulated SLC7A11 to enhance cystine uptake, increases GSH synthesis and counteracted lipid peroxidation; GPX4 utilized GSH as a reducing cofactor to reduce ROS production and mitigate oxidative stress; CHRNA9 promoted Nrf2 nuclear translocation by inhibiting Keap1, thereby activating the antioxidant factor HO-1 and protecting cells from ferroptosis. Therefore, it can be concluded that overexpression of α9 nAChR inhibited the occurrence of ferroptosis in TNBC by promoting the SLC7A11/GSH/GPX4 and Keap1/Nrf2/HO-1 signaling pathways.

Based on above evidence in the present study that α9 nAChR played a positive role in promoting tumor proliferation and resisting ferroptosis in TNBC, we pretty much confirmed its oncogenic character. Moreover, our finding from the CHRNA9 knockdown experiments, conducted both in vitro and in vivo, promisingly suggested that α9 nAChR could represent a novel therapeutic target for drug development against TNBC. Hence, we also evaluated the therapeutic effect of a specific polypeptide antagonist of α9 nAChR, GeXIVA[1,2], in TNBC tumor-bearing mice and achieved a good outcome. We therefore believe that it is promising to develop novel anti-TNBC drugs by targeting α9 nAChR and GeXIVA[1,2] has a great potential for further investigation towards anti-cancer drug development.

Our study demonstrated that overexpression of CHRNA9 significantly promoted the growth of TNBC cells, while knockdown of CHRNA9 markedly suppressed TNBC cell growth. The underlying mechanism uncovered by the present study demonstrated that α9 nAChR was able to activate AKT-, ERK- and STAT3-mediated signaling pathways and suppressed ferroptosis via promoting SLC7A11/GSH/GPX4 and Keap1/Nrf2/HO1 signaling, ultimately supporting the continuous tumor cell growth of TNBC (Figure 7).

## 5. Conclusions

In this study, we demonstrated the oncogenic role of α9 nAChR in regulating tumor proliferation and ferroptosis in TNBC. Firstly, this study uncovered that CHRNA9 expression was elevated in TNBC tissues and was associated with poor prognosis of TNBC patients. Further, this study demonstrated that overexpression of α9 nAChR facilitated the growth of TNBC cells, via mechanisms of simultaneously activating AKT-, ERK- and STAT3-mediated proliferation and negatively regulating ferroptosis through promoting SLC7A11/GSH/GPX4 and Keap1/Nrf2/HO1 signaling. Conversely, CHRNA9 knockdown would completely reverse all this signaling, ultimately inhibiting TNBC tumor growth both in vitro and in vivo. These results together suggested that α9 nAChR is promising to serve as a novel therapeutic target for TNBC. Finally, we reported a specific polypeptide antagonist of α9 nAChR, GeXIVA[1,2], exerted good anti-tumor effects in tumor-bearing mice of TNBC, which indicated a great potential of GeXIVA[1,2] to be further studied as a novel targeted therapy for TNBC.

## Data Availability

The original contributions presented in this study are included in the article/Supplementary Materials. Further inquiries can be directed to the corresponding authors.

## Supplementary Materials

The following supporting information can be downloaded at: https://www.mdpi.com/article/10.3390/biom15060835/s1, File S1. The CHRNA9 expression levels in TNBC tissues (n = 125) and adjacent normal tissues (n = 113) (Source Data for Figure 1A); File S2. The survival time of TNBC patients with high CHRNA9 expression (n = 50) and low CHRNA9 expression (n = 200) (Source Data for Figure 1B); File S3. The densitometric analysis of the overexpression and knockdown efficiencies of α9 nAChR (Source Data for Figure 2A,B); File S4. The cell growth analysis of the overexpression and knockdown effects of α9 nAChR examined by ATP assay (Source Data for Figure 2C–F); File S5. The cell growth analysis of the overexpression and knockdown effects of α9 nAChR examined by colony formation experiment (Source Data for Figure 2G–J); File S6. The densitometric analysis of the overexpression and knockdown effects of α9 nAChR on proliferation and apoptosis pathway (Source Data for Figure 3B–I,K–R); File S7. The quantitative analysis of the overexpression and knockdown effects of α9 nAChR on phenotypic indicators of ferroptosis (Source Data for Figure 4A–L); File S8. The densitometric analysis of the overexpression and knockdown effects of α9 nAChR on ferroptosis pathway (Source Data for Figure 5C–L); File S9. The endpoint tumor weight of HCC38 xenograft nude mice to evaluate anti-tumor effect of α9 nAChR knockdown (Source Data for Figure 6C); File S10. The endpoint tumor weight of 4T1 allograft mice to evaluate anti-tumor effect of α9 nAChR antagonist GeXIVA[1,2] (Source Data for Figure 6F); File S11. Original Western blot images (Source Data for Figure 1C; Figure 2A,B; Figure 3A,J; Figure 5A,B).
