# Supplementary material for: α9 Nicotinic Acetylcholine Receptor Promotes Tumor Proliferation and Suppresses Ferroptosis in Triple-Negative Breast Cancer
Source: Biomolecules. 2025 Jun 8;15(6):835. doi: 10.3390/biom15060835 (PMC12191395; doi:10.3390/biom15060835)

Supplementary Data Files

α9 Nicotinic Acetylcholine Receptor Promotes Tumor Proliferation and Suppresses Ferroptosis in Triple-Negative Breast Cancer

Feng et al.

Description of Supplementary Data Files:

- **File Name: Source Data for Figure 1A**

Description: The CHRNA9 expression levels in TNBC tissues (n=125) and adjacent normal tissues (n=113)

- **File Name: Source Data for Figure 1B**

Description: The survival time of TNBC patients with high CHRNA9 expression (n=50) and low CHRNA9 expression (n=200).

- **File Name: Source Data for Figure 2A&B**

Description: The densitometric analysis of the overexpression and knockdown efficiencies of α9 nAChR.

- **File Name: Source Data for Figure 2C-F**

Description: The cell growth analysis of the overexpression and knockdown effects of α9 nAChR examined by ATP assay.

- **File Name: Source Data for Figure 2G-J**

Description: The cell growth analysis of the overexpression and knockdown effects of α9 nAChR examined by colony formation experiment.

- **File Name: Source Data for Figure 3B-I, 3K-R**

Description: The densitometric analysis of the overexpression and knockdown effects of α9 nAChR on proliferation and apoptosis pathway.

- **File Name: Source Data for Figure 4A-L**

Description: The quantitative analysis of the overexpression and knockdown effects of α9 nAChR on phenotypic indicators of ferroptosis.

- **File Name: Source Data for Figure 5C-L**

Description: The densitometric analysis of the overexpression and knockdown effects of α9 nAChR on ferroptosis pathway.

- **File Name: Source Data for Figure 6B**

Description: The endpoint tumor weight of HCC38 xenograft nude mice to evaluate anti-tumor effect of α9 nAChR knockdown.

- **File Name: Source Data for Figure 6E**

Description: The endpoint tumor weight of 4T1 allograft mice to evaluate anti-tumor effect of α9 nAChR antagonist GeXIVA[1,2].

- **File Name: Source Data for Western Blot Images**

## Source Data for Figure 1A

The CHRNA9 expression levels in TNBC tissues (n=125) and adjacent normal tissues (n=113)

| | sample_id | CHRNA9 | Group |
|---|---|---|---|
| 1 | TCGA-A2-A04U-01 | 0 | Tumor |
| 2 | TCGA-E2-A1LG-01 | 0.0142 | Tumor |
| 3 | TCGA-BH-A0DP-11 | 0.0997 | Normal |
| 4 | TCGA-EW-A1P8-01 | 0.0205 | Tumor |
| 5 | TCGA-BH-A18G-01 | 0 | Tumor |
| 6 | TCGA-E2-A1LB-11 | 0.1709 | Normal |
| 7 | TCGA-BH-A1FU-11 | 0 | Normal |
| 8 | TCGA-E9-A1ND-01 | 0.1746 | Tumor |
| 9 | TCGA-E9-A1ND-11 | 0.0202 | Normal |
| 10 | TCGA-AR-A1AR-01 | 0 | Tumor |
| 11 | TCGA-A7-A0DB-11 | 0.0656 | Normal |
| 12 | TCGA-BH-A0DK-11 | 0.0648 | Normal |
| 13 | TCGA-E9-A1RF-11 | 0 | Normal |
| 14 | TCGA-AO-A129-01 | 0.1731 | Tumor |
| 15 | TCGA-AN-A0AR-01 | 0 | Tumor |
| 16 | TCGA-A7-A26I-01 | 0.1322 | Tumor |
| 17 | TCGA-A8-A07C-01 | 0.0602 | Tumor |
| 18 | TCGA-BH-A18R-11 | 0.0157 | Normal |
| 19 | TCGA-EW-A1P1-01 | 0.0273 | Tumor |
| 20 | TCGA-GI-A2C8-11 | 0 | Normal |
| 21 | TCGA-BH-A0C0-11 | 0 | Normal |
| 22 | TCGA-B6-A0IK-01 | 0.1902 | Tumor |
| 23 | TCGA-E2-A1L7-01 | 0.1929 | Tumor |
| 24 | TCGA-AR-A0TS-01 | 0.0405 | Tumor |
| 25 | TCGA-E2-A1IG-11 | 0 | Normal |
| 26 | TCGA-E9-A1NA-11 | 0 | Normal |
| 27 | TCGA-AC-A2FF-11 | 0.1344 | Normal |
| 28 | TCGA-BH-A0BW-11 | 0.1283 | Normal |
| 29 | TCGA-AN-A0FL-01 | 0.032 | Tumor |
| 30 | TCGA-A1-A0SP-01 | 0.0565 | Tumor |
| 31 | TCGA-BH-A1F8-11 | 0 | Normal |
| 32 | TCGA-BH-A0BQ-11 | 0.0501 | Normal |
| 33 | TCGA-A1-A0SK-01 | 3.2349 | Tumor |
| 34 | TCGA-A2-A04Q-01 | 0.0676 | Tumor |
| 35 | TCGA-BH-A0DT-11 | 0.0219 | Normal |
| 36 | TCGA-E2-A14N-01 | 0 | Tumor |
| 37 | TCGA-A1-A0SO-01 | 0 | Tumor |

| | | | |
|---|---|---|---|
| 38 | TCGA-BH-A18N-11 | 0.0186 | Normal |
| 39 | TCGA-E9-A1N4-11 | 0.0681 | Normal |
| 40 | TCGA-A7-A13G-11 | 0 | Normal |
| 41 | TCGA-AC-A2FB-11 | 0.0432 | Normal |
| 42 | TCGA-BH-A0DQ-11 | 0.0171 | Normal |
| 43 | TCGA-D8-A143-01 | 0.0636 | Tumor |
| 44 | TCGA-BH-A1FD-11 | 0.0156 | Normal |
| 45 | TCGA-BH-A0HA-11 | 0.0242 | Normal |
| 46 | TCGA-BH-A0BM-11 | 0 | Normal |
| 47 | TCGA-BH-A1FE-11 | 0.0385 | Normal |
| 48 | TCGA-C8-A12V-01 | 0.1937 | Tumor |
| 49 | TCGA-BH-A1EN-11 | 0.0256 | Normal |
| 50 | TCGA-A7-A26F-01 | 0.1559 | Tumor |
| 51 | TCGA-A7-A26G-01 | 0.0672 | Tumor |
| 52 | TCGA-BH-A0B5-11 | 0 | Normal |
| 53 | TCGA-E2-A15I-11 | 0 | Normal |
| 54 | TCGA-C8-A26X-01 | 0.082 | Tumor |
| 55 | TCGA-A7-A0DC-11 | 4.2805 | Normal |
| 56 | TCGA-A7-A0CH-11 | 0.1428 | Normal |
| 57 | TCGA-A2-A0D2-01 | 0.1431 | Tumor |
| 58 | TCGA-B6-A0RG-01 | 0.1603 | Tumor |
| 59 | TCGA-BH-A1EW-11 | 0.0402 | Normal |
| 60 | TCGA-BH-A204-11 | 0.5143 | Normal |
| 61 | TCGA-AN-A0AT-01 | 0.0147 | Tumor |
| 62 | TCGA-E2-A1L7-11 | 0.0724 | Normal |
| 63 | TCGA-AR-A0U0-01 | 0.0206 | Tumor |
| 64 | TCGA-A2-A0YM-01 | 0.0794 | Tumor |
| 65 | TCGA-BH-A18L-11 | 0.046 | Normal |
| 66 | TCGA-C8-A134-01 | 0.0522 | Tumor |
| 67 | TCGA-D8-A1JG-01 | 0.0354 | Tumor |
| 68 | TCGA-BH-A0BZ-11 | 0 | Normal |
| 69 | TCGA-B6-A0RT-01 | 0.1613 | Tumor |
| 70 | TCGA-B6-A0WX-01 | 0 | Tumor |
| 71 | TCGA-BH-A18T-01 | 0.0552 | Tumor |
| 72 | TCGA-BH-A209-11 | 0.0321 | Normal |
| 73 | TCGA-BH-A0BL-01 | 1.5835 | Tumor |
| 74 | TCGA-E9-A1N6-11 | 0.1788 | Normal |
| 75 | TCGA-B6-A0IQ-01 | 0.2839 | Tumor |
| 76 | TCGA-AN-A0XU-01 | 6.5964 | Tumor |
| 77 | TCGA-AN-A0AL-01 | 0.0921 | Tumor |
| 78 | TCGA-E2-A1LI-01 | 0.0132 | Tumor |
| 79 | TCGA-S3-AA11-01 | 0.1135 | Tumor |

| | | | |
|---|---|---|---|
| 80 | TCGA-AO-A124-01 | 0.0505 | Tumor |
| 81 | TCGA-AO-A128-01 | 0.033 | Tumor |
| 82 | TCGA-BH-A1FB-11 | 0 | Normal |
| 83 | TCGA-A7-A26F-01.1 | 0.0724 | Tumor |
| 84 | TCGA-B6-A0IE-01 | 0.1568 | Tumor |
| 85 | TCGA-C8-A131-01 | 0.7216 | Tumor |
| 86 | TCGA-E9-A1RI-11 | 0 | Normal |
| 87 | TCGA-BH-A0BV-11 | 0.0561 | Normal |
| 88 | TCGA-AR-A0U4-01 | 0.3325 | Tumor |
| 89 | TCGA-A7-A0CE-11 | 0.067 | Normal |
| 90 | TCGA-BH-A1EW-01 | 0.2153 | Tumor |
| 91 | TCGA-A7-A0CE-01 | 0.8258 | Tumor |
| 92 | TCGA-B6-A0RU-01 | 0.0214 | Tumor |
| 93 | TCGA-BH-A18V-11 | 0 | Normal |
| 94 | TCGA-EW-A1P7-01 | 0.2443 | Tumor |
| 95 | TCGA-C8-A1HJ-01 | 0.1798 | Tumor |
| 96 | TCGA-A7-A0DA-01 | 0.0877 | Tumor |
| 97 | TCGA-E9-A22G-01 | 0.0289 | Tumor |
| 98 | TCGA-BH-A0BS-11 | 0 | Normal |
| 99 | TCGA-BH-A0BW-01 | 0.313 | Tumor |
| 100 | TCGA-E9-A1NF-11 | 0.024 | Normal |
| 101 | TCGA-BH-A0H9-11 | 0 | Normal |
| 102 | TCGA-BH-A1F6-11 | 0.0814 | Normal |
| 103 | TCGA-A2-A0T2-01 | 0.1605 | Tumor |
| 104 | TCGA-BH-A1F2-11 | 0.0363 | Normal |
| 105 | TCGA-BH-A18V-01 | 0.0427 | Tumor |
| 106 | TCGA-BH-A0E6-01 | 0 | Tumor |
| 107 | TCGA-BH-A0B7-11 | 0.0526 | Normal |
| 108 | TCGA-E9-A1NG-11 | 0.0659 | Normal |
| 109 | TCGA-BH-A0B8-11 | 0 | Normal |
| 110 | TCGA-BH-A0BJ-11 | 0.0252 | Normal |
| 111 | TCGA-BH-A0DG-11 | 0.0403 | Normal |
| 112 | TCGA-D8-A1JF-01 | 0.0901 | Tumor |
| 113 | TCGA-BH-A0DD-11 | 0.018 | Normal |
| 114 | TCGA-E9-A1R7-11 | 0.0173 | Normal |
| 115 | TCGA-BH-A208-11 | 0 | Normal |
| 116 | TCGA-BH-A0WA-01 | 0 | Tumor |
| 117 | TCGA-D8-A13Z-01 | 0.0333 | Tumor |
| 118 | TCGA-BH-A0B9-01 | 0.0364 | Tumor |
| 119 | TCGA-B6-A0RS-01 | 0.042 | Tumor |
| 120 | TCGA-A2-A0T0-01 | 0.0165 | Tumor |
| 121 | TCGA-E9-A1RB-11 | 0.0272 | Normal |

| | | | |
|---|---|---|---|
| 122 | TCGA-D8-A1XK-01 | 0.1937 | Tumor |
| 123 | TCGA-BH-A0AU-11 | 0.0243 | Normal |
| 124 | TCGA-D8-A1XQ-01 | 0 | Tumor |
| 125 | TCGA-E9-A1RD-11 | 0 | Normal |
| 126 | TCGA-BH-A0E1-11 | 0.0254 | Normal |
| 127 | TCGA-E2-A14X-01 | 0.049 | Tumor |
| 128 | TCGA-E9-A1RH-11 | 0.0274 | Normal |
| 129 | TCGA-BH-A1FC-01 | 0.0235 | Tumor |
| 130 | TCGA-B6-A0RE-01 | 0.0209 | Tumor |
| 131 | TCGA-A2-A0SX-01 | 0.0824 | Tumor |
| 132 | TCGA-BH-A0DO-11 | 0.0412 | Normal |
| 133 | TCGA-A8-A09X-01 | 0.0371 | Tumor |
| 134 | TCGA-BH-A0C3-11 | 0.0614 | Normal |
| 135 | TCGA-E9-A1RC-11 | 0.0125 | Normal |
| 136 | TCGA-AN-A0G0-01 | 0.1711 | Tumor |
| 137 | TCGA-AR-A1AY-01 | 0.0962 | Tumor |
| 138 | TCGA-A8-A08R-01 | 0.0158 | Tumor |
| 139 | TCGA-BH-A1EV-11 | 0.0266 | Normal |
| 140 | TCGA-BH-A1FC-11 | 0.0943 | Normal |
| 141 | TCGA-BH-A0E0-11 | 0 | Normal |
| 142 | TCGA-E2-A1LL-01 | 0.3721 | Tumor |
| 143 | TCGA-E2-A1BC-11 | 0.0703 | Normal |
| 144 | TCGA-BH-A18Q-11 | 0.1355 | Normal |
| 145 | TCGA-E2-A15M-11 | 0 | Normal |
| 146 | TCGA-A7-A13E-11 | 0 | Normal |
| 147 | TCGA-D8-A142-01 | 0.3501 | Tumor |
| 148 | TCGA-BH-A0DH-11 | 0 | Normal |
| 149 | TCGA-E2-A1B6-01 | 0.1346 | Tumor |
| 150 | TCGA-E2-A1LS-01 | 0.009 | Tumor |
| 151 | TCGA-E2-A1LS-11 | 0.0108 | Normal |
| 152 | TCGA-BH-A0AY-11 | 0 | Normal |
| 153 | TCGA-BH-A0DV-11 | 0.0199 | Normal |
| 154 | TCGA-BH-A0H5-11 | 0 | Normal |
| 155 | TCGA-AC-A2FM-11 | 0.0379 | Normal |
| 156 | TCGA-BH-A18M-11 | 0.0527 | Normal |
| 157 | TCGA-AO-A0J4-01 | 0.1345 | Tumor |
| 158 | TCGA-D8-A1JL-01 | 0.1664 | Tumor |
| 159 | TCGA-BH-A0RX-01 | 0.0537 | Tumor |
| 160 | TCGA-D8-A27M-01 | 0.0851 | Tumor |
| 161 | TCGA-EW-A1OV-01 | 0.0216 | Tumor |
| 162 | TCGA-EW-A1PB-01 | 0.0173 | Tumor |
| 163 | TCGA-BH-A203-11 | 0 | Normal |

| | | | |
|---|---|---|---|
| 164 | TCGA-AO-A0J6-01 | 0.0254 | Tumor |
| 165 | TCGA-A2-A0CM-01 | 0.0505 | Tumor |
| 166 | TCGA-E2-A158-01 | 0.0172 | Tumor |
| 167 | TCGA-A2-A0D0-01 | 0.2025 | Tumor |
| 168 | TCGA-AN-A04D-01 | 0.0139 | Tumor |
| 169 | TCGA-E2-A150-01 | 0 | Tumor |
| 170 | TCGA-E9-A1N5-11 | 0.0433 | Normal |
| 171 | TCGA-BH-A1ET-11 | 0.0309 | Normal |
| 172 | TCGA-AR-A1AI-01 | 0 | Tumor |
| 173 | TCGA-BH-A18J-11 | 0 | Normal |
| 174 | TCGA-E9-A1N9-11 | 0.0379 | Normal |
| 175 | TCGA-BH-A0HK-11 | 0 | Normal |
| 176 | TCGA-A7-A26I-01.1 | 0.0887 | Tumor |
| 177 | TCGA-EW-A1OW-01 | 0.2115 | Tumor |
| 178 | TCGA-D8-A147-01 | 0.0226 | Tumor |
| 179 | TCGA-E2-A158-11 | 0 | Normal |
| 180 | TCGA-BH-A1FR-11 | 0 | Normal |
| 181 | TCGA-A2-A0YE-01 | 0.1976 | Tumor |
| 182 | TCGA-BH-A0BC-11 | 0 | Normal |
| 183 | TCGA-BH-A0BA-11 | 0.1245 | Normal |
| 184 | TCGA-AR-A1AQ-01 | 0.2382 | Tumor |
| 185 | TCGA-AQ-A04J-01 | 0.1211 | Tumor |
| 186 | TCGA-A2-A04T-01 | 0 | Tumor |
| 187 | TCGA-E2-A1AZ-01 | 0.1784 | Tumor |
| 188 | TCGA-BH-A1FJ-11 | 0.0587 | Normal |
| 189 | TCGA-C8-A27B-01 | 0 | Tumor |
| 190 | TCGA-B6-A0RN-01 | 2.3589 | Tumor |
| 191 | TCGA-BH-A1EU-11 | 0 | Normal |
| 192 | TCGA-GI-A2C9-11 | 0.0185 | Normal |
| 193 | TCGA-E2-A1LH-01 | 0.0192 | Tumor |
| 194 | TCGA-AO-A0J2-01 | 0.0138 | Tumor |
| 195 | TCGA-BH-A1FG-11 | 0.3218 | Normal |
| 196 | TCGA-BH-A18Q-01 | 0.0259 | Tumor |
| 197 | TCGA-BH-A1FN-11 | 0.0421 | Normal |
| 198 | TCGA-D8-A27H-01 | 0.1164 | Tumor |
| 199 | TCGA-BH-A0DL-11 | 0.0348 | Normal |
| 200 | TCGA-BH-A0AV-01 | 0.4231 | Tumor |
| 201 | TCGA-BH-A1F0-11 | 0.046 | Normal |
| 202 | TCGA-BH-A18U-11 | 0 | Normal |
| 203 | TCGA-BH-A0BT-11 | 0.124 | Normal |
| 204 | TCGA-E2-A1LK-01 | 0 | Tumor |
| 205 | TCGA-BH-A0BG-01 | 0 | Tumor |

| | | | |
|---|---|---|---|
| 206 | TCGA-E2-A14R-01 | 0.0165 | Tumor |
| 207 | TCGA-BH-A18K-11 | 0 | Normal |
| 208 | TCGA-BH-A0AZ-11 | 0 | Normal |
| 209 | TCGA-EW-A1P4-01 | 0.1821 | Tumor |
| 210 | TCGA-A2-A0ST-01 | 0 | Tumor |
| 211 | TCGA-AN-A0FX-01 | 0.0556 | Tumor |
| 212 | TCGA-A7-A13F-11 | 0.0208 | Normal |
| 213 | TCGA-BH-A1FH-11 | 0.0284 | Normal |
| 214 | TCGA-BH-A0E0-01 | 4.5199 | Tumor |
| 215 | TCGA-AO-A03U-01 | 0.0721 | Tumor |
| 216 | TCGA-BH-A0B3-11 | 0.0819 | Normal |
| 217 | TCGA-A7-A0D9-11 | 0 | Normal |
| 218 | TCGA-BH-A18P-11 | 0 | Normal |
| 219 | TCGA-E2-A1LH-11 | 0.0579 | Normal |
| 220 | TCGA-E2-A159-01 | 0.2343 | Tumor |
| 221 | TCGA-E2-A153-11 | 0.1013 | Normal |
| 222 | TCGA-C8-A26Y-01 | 0.0557 | Tumor |
| 223 | TCGA-BH-A1FM-11 | 0.0323 | Normal |
| 224 | TCGA-BH-A18S-11 | 0.0513 | Normal |
| 225 | TCGA-BH-A0DZ-11 | 0.0232 | Normal |
| 226 | TCGA-A2-A04P-01 | 0 | Tumor |
| 227 | TCGA-D8-A27F-01 | 0.1854 | Tumor |
| 228 | TCGA-BH-A0H7-11 | 0 | Normal |
| 229 | TCGA-BH-A0B3-01 | 0.0834 | Tumor |
| 230 | TCGA-E2-A15K-11 | 0.0165 | Normal |
| 231 | TCGA-AO-A0JL-01 | 0.1 | Tumor |
| 232 | TCGA-AR-A256-01 | 0.0374 | Tumor |
| 233 | TCGA-A2-A1G6-01 | 0.0209 | Tumor |
| 234 | TCGA-BH-A1EO-11 | 0 | Normal |
| 235 | TCGA-AC-A23H-11 | 0.0438 | Normal |
| 236 | TCGA-AO-A12F-01 | 0.129 | Tumor |
| 237 | TCGA-A8-A07O-01 | 0 | Tumor |
| 238 | TCGA-AR-A0TU-01 | 0 | Tumor |

## Source Data for Figure 1B

The survival time of TNBC patients with high CHRNA9 expression (n=50) and low CHRNA9 expression (n=200).

| Patient ID | CHRNA9-z-Score | ER Status | HER2 Status | PR Status | Overall Survival (Months) | Patient's Vital Status |
|---|---|---|---|---|---|---|
| MB-0062 | 0.5276 | Negative | Negative | Negative | 153.966667 | Living |
| MB-0079 | 0.1788 | Negative | Negative | Negative | 28.5 | Died of Disease |
| MB-0100 | -0.2787 | Negative | Negative | Negative | 8.06666667 | Died of Disease |
| MB-0115 | -0.0916 | Negative | Negative | Negative | 66.7333333 | Died of Disease |
| MB-0127 | 0.1438 | Negative | Negative | Negative | 132.066667 | Living |
| MB-0157 | -0.459 | Negative | Negative | Negative | 114.766667 | Living |
| MB-0164 | -0.3841 | Negative | Negative | Negative | 10.8333333 | Living |
| MB-0174 | -0.3253 | Negative | Negative | Negative | 78.7666667 | Living |
| MB-0179 | 0.2073 | Negative | Negative | Negative | 17.9333333 | Died of Disease |
| MB-0188 | -0.1587 | Negative | Negative | Negative | 31.3 | Died of Disease |
| MB-0200 | -0.1425 | Negative | Negative | Negative | 130.866667 | Died of Disease |
| MB-0206 | -0.4698 | Negative | Negative | Negative | 141.166667 | Living |
| MB-0211 | 0.2838 | Negative | Negative | Negative | 44.8 | Died of Disease |
| MB-0214 | -0.1878 | Negative | Negative | Negative | 146.9 | Living |
| MB-0220 | 0.0003 | Negative | Negative | Negative | 39.8333333 | Died of Disease |
| MB-0221 | -0.3832 | Negative | Negative | Negative | 20.2 | Died of Disease |
| MB-0238 | -0.3274 | Negative | Negative | Negative | 193.166667 | Living |
| MB-0249 | 0.0317 | Negative | Negative | Negative | 188.333333 | Living |
| MB-0259 | 0.2112 | Negative | Negative | Negative | 11.8666667 | Died of Disease |
| MB-0265 | -0.0251 | Negative | Negative | Negative | 189.133333 | Living |
| MB-0269 | -0.2432 | Negative | Negative | Negative | 22.2333333 | Living |
| MB-0278 | 0.1971 | Negative | Negative | Negative | 5.83333333 | Died of Disease |
| MB-0294 | -0.1374 | Negative | Negative | Negative | 72.9 | Living |
| MB-0303 | -0.3806 | Negative | Negative | Negative | 60.1333333 | Living |
| MB-0316 | -0.0123 | Negative | Negative | Negative | 182.9 | Living |
| MB-0318 | -0.1891 | Negative | Negative | Negative | 168.7 | Living |
| MB-0340 | -0.4088 | Negative | Negative | Negative | 164.733333 | Living |
| MB-0350 | -0.3645 | Negative | Negative | Negative | 46.0666667 | Died of Disease |
| MB-0352 | -0.1227 | Negative | Negative | Negative | 55.2 | Died of Disease |
| MB-0354 | -0.268 | Negative | Negative | Negative | 11.6 | Died of Disease |
| MB-0372 | -0.0858 | Negative | Negative | Negative | 62.7666667 | Died of Disease |
| MB-0396 | -0.411 | Negative | Negative | Negative | 60.6666667 | Living |
| MB-0400 | 0.0041 | Negative | Negative | Negative | 22.4666667 | Died of Disease |
| MB-0401 | -0.0337 | Negative | Negative | Negative | 26.2666667 | Died of Disease |
| MB-0414 | -0.0449 | Negative | Negative | Negative | 76.6333333 | Living |

| | | | | | | |
|---|---|---|---|---|---|---|
| MB-0420 | -0.5777 | Negative | Negative | Negative | 76.7333333 | Living |
| MB-0424 | -0.1052 | Negative | Negative | Negative | 9.83333333 | Died of Disease |
| MB-0446 | -0.3262 | Negative | Negative | Negative | 72.2666667 | Living |
| MB-0453 | -0.1008 | Negative | Negative | Negative | 43.1333333 | Died of Disease |
| MB-0464 | -0.2147 | Negative | Negative | Negative | 39.3 | Died of Disease |
| MB-0470 | -0.1266 | Negative | Negative | Negative | 88.2333333 | Living |
| MB-0481 | -0.0219 | Negative | Negative | Negative | 36.4 | Died of Disease |
| MB-0489 | -0.0899 | Negative | Negative | Negative | 90.5666667 | Living |
| MB-0494 | -0.127 | Negative | Negative | Negative | 36.6333333 | Died of Disease |
| MB-0495 | -0.6308 | Negative | Negative | Negative | 71.8 | Died of Disease |
| MB-0500 | -0.6988 | Negative | Negative | Negative | 67.4666667 | Living |
| MB-0502 | 0.1053 | Negative | Negative | Negative | 82.1 | Living |
| MB-0516 | -0.7893 | Negative | Negative | Negative | 114.466667 | Living |
| MB-0525 | -0.0269 | Negative | Negative | Negative | 65.8666667 | Living |
| MB-0540 | -0.009 | Negative | Negative | Negative | 194.533333 | Living |
| MB-0581 | 0.0994 | Negative | Negative | Negative | 7.86666667 | Died of Disease |
| MB-0588 | 0.2662 | Negative | Negative | Negative | 119.733333 | Living |
| MB-0613 | 0.4319 | Negative | Negative | Negative | 48.8 | Living |
| MB-0627 | -0.0922 | Negative | Negative | Negative | 0.76666667 | Living |
| MB-0634 | 0.0054 | Negative | Negative | Negative | 17.7666667 | Living |
| MB-0639 | -0.404 | Negative | Negative | Negative | 75.4 | Living |
| MB-0653 | 0.1578 | Negative | Negative | Negative | 24.8 | Died of Disease |
| MB-0658 | -0.0283 | Negative | Negative | Negative | 97.2666667 | Living |
| MB-0659 | -0.1533 | Negative | Negative | Negative | 23.3333333 | Died of Disease |
| MB-0660 | 0.2071 | Negative | Negative | Negative | 18.9333333 | Died of Disease |
| MB-0664 | -0.399 | Negative | Negative | Negative | 11.0666667 | Died of Disease |
| MB-0874 | 0.3108 | Negative | Negative | Negative | 16.6 | Died of Disease |
| MB-0893 | -0.4116 | Negative | Negative | Negative | 175.633333 | Living |
| MB-0901 | 0.2529 | Negative | Negative | Negative | 136.166667 | Living |
| MB-2643 | -0.4494 | Negative | Negative | Negative | 52.9666667 | Died of Disease |
| MB-2718 | -0.0142 | Negative | Negative | Negative | 278.266667 | Living |
| MB-2724 | -0.3465 | Negative | Negative | Negative | 108.066667 | Died of Disease |
| MB-2753 | 0.1901 | Negative | Negative | Negative | 274.4 | Living |
| MB-2754 | -0.1931 | Negative | Negative | Negative | 203.533333 | Died of Disease |
| MB-2771 | -0.2923 | Negative | Negative | Negative | 44.8333333 | Died of Disease |
| MB-2827 | -0.2779 | Negative | Negative | Negative | 235.666667 | Living |
| MB-2833 | -0.0114 | Negative | Negative | Negative | 259.766667 | Living |
| MB-2842 | 0.0969 | Negative | Negative | Negative | 19.7333333 | Died of Disease |
| MB-2849 | -0.4018 | Negative | Negative | Negative | 43.2 | Died of Disease |
| MB-2850 | -0.0882 | Negative | Negative | Negative | 9.43333333 | Died of Disease |
| MB-2857 | -0.3102 | Negative | Negative | Negative | 250.666667 | Living |
| MB-2904 | -0.5214 | Negative | Negative | Negative | 125.6 | Died of Disease |

| | | | | | | |
|---|---|---|---|---|---|---|
| MB-2912 | -0.14 | Negative | Negative | Negative | 267.4 | Living |
| MB-2917 | -0.3178 | Negative | Negative | Negative | 31.9333333 | Died of Disease |
| MB-2922 | -0.3506 | Negative | Negative | Negative | 14.4 | Died of Disease |
| MB-2957 | 0.0892 | Negative | Negative | Negative | 262.133333 | Living |
| MB-2963 | -0.2591 | Negative | Negative | Negative | 227.833333 | Died of Disease |
| MB-2993 | -0.3714 | Negative | Negative | Negative | 187.033333 | Living |
| MB-3001 | -0.0128 | Negative | Negative | Negative | 263.233333 | Living |
| MB-3014 | -0.1983 | Negative | Negative | Negative | 262.633333 | Living |
| MB-3057 | -0.3097 | Negative | Negative | Negative | 32.0333333 | Died of Disease |
| MB-3058 | -0.3327 | Negative | Negative | Negative | 254.266667 | Living |
| MB-3062 | -0.3648 | Negative | Negative | Negative | 146.366667 | Living |
| MB-3063 | -0.1068 | Negative | Negative | Negative | 28.5666667 | Died of Disease |
| MB-3067 | -0.1156 | Negative | Negative | Negative | 259.533333 | Living |
| MB-3123 | 0.392 | Negative | Negative | Negative | 167.433333 | Living |
| MB-3153 | -0.3448 | Negative | Negative | Negative | 227.933333 | Living |
| MB-3211 | -0.0892 | Negative | Negative | Negative | 145.5 | Living |
| MB-3218 | -0.0726 | Negative | Negative | Negative | 248.766667 | Living |
| MB-3277 | -0.5109 | Negative | Negative | Negative | 32.9333333 | Died of Disease |
| MB-3292 | -0.2415 | Negative | Negative | Negative | 217.766667 | Living |
| MB-3297 | -0.3076 | Negative | Negative | Negative | 236.066667 | Living |
| MB-3383 | 0.9645 | Negative | Negative | Negative | 23.2 | Died of Disease |
| MB-3395 | -0.3083 | Negative | Negative | Negative | 243.766667 | Living |
| MB-3396 | -0.4068 | Negative | Negative | Negative | 226.733333 | Living |
| MB-3453 | -0.118 | Negative | Negative | Negative | 32.8333333 | Died of Disease |
| MB-3500 | -0.2315 | Negative | Negative | Negative | 239.3 | Living |
| MB-3502 | -0.0711 | Negative | Negative | Negative | 229.333333 | Living |
| MB-3567 | 0.1755 | Negative | Negative | Negative | 236.033333 | Living |
| MB-3702 | -0.2698 | Negative | Negative | Negative | 230.466667 | Living |
| MB-3706 | -0.2614 | Negative | Negative | Negative | 38.8 | Died of Disease |
| MB-3752 | -0.1864 | Negative | Negative | Negative | 75.3333333 | Died of Disease |
| MB-4015 | -0.3213 | Negative | Negative | Negative | 11.3 | Died of Disease |
| MB-4024 | -0.1638 | Negative | Negative | Negative | 124.1 | Living |
| MB-4146 | -0.3087 | Negative | Negative | Negative | 15.6333333 | Died of Disease |
| MB-4254 | -0.5522 | Negative | Negative | Negative | 178.4 | Living |
| MB-4332 | 0.025 | Negative | Negative | Negative | 307.933333 | Living |
| MB-4351 | -0.4336 | Negative | Negative | Negative | 68.1333333 | Died of Disease |
| MB-4354 | -0.0221 | Negative | Negative | Negative | 15.3666667 | Died of Disease |
| MB-4407 | 0.3206 | Negative | Negative | Negative | 83.3666667 | Died of Disease |
| MB-4416 | -0.3689 | Negative | Negative | Negative | 241.6 | Died of Disease |
| MB-4621 | -0.3522 | Negative | Negative | Negative | 271.866667 | Living |
| MB-4640 | -0.3396 | Negative | Negative | Negative | 200.6 | Living |
| MB-4660 | -0.4898 | Negative | Negative | Negative | 39.8666667 | Died of Disease |

| | | | | | | |
|---|---|---|---|---|---|---|
| MB-4696 | 6.0112 | Negative | Negative | Negative | 255.266667 | Living |
| MB-4707 | -0.23 | Negative | Negative | Negative | 221.2 | Living |
| MB-4715 | -0.446 | Negative | Negative | Negative | 35.2333333 | Died of Disease |
| MB-4717 | -0.0611 | Negative | Negative | Negative | 42.9 | Died of Disease |
| MB-4732 | -0.6189 | Negative | Negative | Negative | 149.866667 | Living |
| MB-4733 | -0.2858 | Negative | Negative | Negative | 19.7333333 | Died of Disease |
| MB-4757 | -0.2443 | Negative | Negative | Negative | 27.4 | Died of Disease |
| MB-4758 | -0.1282 | Negative | Negative | Negative | 211.933333 | Living |
| MB-4769 | -0.3537 | Negative | Negative | Negative | 21.7 | Died of Disease |
| MB-4792 | 0.0973 | Negative | Negative | Negative | 40.6333333 | Died of Disease |
| MB-4865 | 0.0696 | Negative | Negative | Negative | 229.9 | Living |
| MB-4880 | -0.3505 | Negative | Negative | Negative | 210.966667 | Living |
| MB-4881 | -0.4842 | Negative | Negative | Negative | 274.2 | Living |
| MB-4888 | -0.273 | Negative | Negative | Negative | 230.5 | Living |
| MB-4893 | -0.3321 | Negative | Negative | Negative | 176.7 | Living |
| MB-4911 | -0.0612 | Negative | Negative | Negative | 31.4333333 | Died of Disease |
| MB-4928 | 0.1134 | Negative | Negative | Negative | 220.033333 | Living |
| MB-4931 | -0.2538 | Negative | Negative | Negative | 30.3666667 | Died of Disease |
| MB-4938 | -0.0522 | Negative | Negative | Negative | 70.6 | Died of Disease |
| MB-4945 | -0.4251 | Negative | Negative | Negative | 20.1333333 | Died of Disease |
| MB-4982 | 0.304 | Negative | Negative | Negative | 19.1666667 | Died of Disease |
| MB-4992 | -0.3713 | Negative | Negative | Negative | 165.366667 | Living |
| MB-4993 | -0.3989 | Negative | Negative | Negative | 75.2333333 | Died of Disease |
| MB-5008 | 0.1503 | Negative | Negative | Negative | 223.3 | Living |
| MB-5041 | -0.1883 | Negative | Negative | Negative | 173.933333 | Living |
| MB-5057 | -0.3591 | Negative | Negative | Negative | 56.3333333 | Died of Disease |
| MB-5058 | -0.3823 | Negative | Negative | Negative | 178.733333 | Died of Disease |
| MB-5065 | -0.2902 | Negative | Negative | Negative | 184.8 | Living |
| MB-5070 | 0.1504 | Negative | Negative | Negative | 213.9 | Living |
| MB-5072 | -0.0112 | Negative | Negative | Negative | 50.2333333 | Died of Disease |
| MB-5100 | -0.3808 | Negative | Negative | Negative | 19.9 | Died of Disease |
| MB-5102 | -0.379 | Negative | Negative | Negative | 30.3666667 | Died of Disease |
| MB-5115 | -0.2111 | Negative | Negative | Negative | 77.4 | Living |
| MB-5126 | -0.0134 | Negative | Negative | Negative | 27.3 | Died of Disease |
| MB-5135 | -0.2231 | Negative | Negative | Negative | 37.3666667 | Died of Disease |
| MB-5138 | -0.5071 | Negative | Negative | Negative | 37 | Died of Disease |
| MB-5145 | 0.0194 | Negative | Negative | Negative | 159.233333 | Died of Disease |
| MB-5155 | -0.033 | Negative | Negative | Negative | 259.933333 | Living |
| MB-5162 | -0.3239 | Negative | Negative | Negative | 164.333333 | Living |
| MB-5173 | -0.1396 | Negative | Negative | Negative | 22.1333333 | Died of Disease |
| MB-5208 | -0.1315 | Negative | Negative | Negative | 151.9 | Living |
| MB-5213 | -0.1553 | Negative | Negative | Negative | 43.4 | Died of Disease |

| | | | | | | |
|---|---|---|---|---|---|---|
| MB-5223 | -0.1571 | Negative | Negative | Negative | 144.666667 | Died of Disease |
| MB-5225 | -0.1569 | Negative | Negative | Negative | 43.2666667 | Died of Disease |
| MB-5235 | -0.6247 | Negative | Negative | Negative | 204.2 | Died of Disease |
| MB-5236 | -0.0655 | Negative | Negative | Negative | 166.666667 | Living |
| MB-5281 | -0.0845 | Negative | Negative | Negative | 79.3 | Living |
| MB-5298 | 0.0633 | Negative | Negative | Negative | 84.9 | Died of Disease |
| MB-5299 | -0.1943 | Negative | Negative | Negative | 16.7 | Died of Disease |
| MB-5323 | -0.1688 | Negative | Negative | Negative | 226.066667 | Living |
| MB-5335 | 0.3295 | Negative | Negative | Negative | 25.2333333 | Died of Disease |
| MB-5346 | -0.1599 | Negative | Negative | Negative | 194.3 | Living |
| MB-5348 | 0.0751 | Negative | Negative | Negative | 169.666667 | Living |
| MB-5390 | 0.2913 | Negative | Negative | Negative | 30.9333333 | Died of Disease |
| MB-5392 | -0.1285 | Negative | Negative | Negative | 237.266667 | Living |
| MB-5408 | 0.0497 | Negative | Negative | Negative | 101.4 | Living |
| MB-5421 | -0.557 | Negative | Negative | Negative | 194.566667 | Living |
| MB-5440 | -0.3014 | Negative | Negative | Negative | 196.633333 | Living |
| MB-5446 | -0.0634 | Negative | Negative | Negative | 162.833333 | Died of Disease |
| MB-5450 | -0.299 | Negative | Negative | Negative | 190.2 | Living |
| MB-5465 | -0.5533 | Negative | Negative | Negative | 18.8 | Died of Disease |
| MB-5526 | -0.0503 | Negative | Negative | Negative | 151.066667 | Living |
| MB-5529 | -0.0105 | Negative | Negative | Negative | 14.8 | Died of Disease |
| MB-5531 | -0.5974 | Negative | Negative | Negative | 183.966667 | Living |
| MB-5547 | -0.2892 | Negative | Negative | Negative | 98.5666667 | Living |
| MB-5548 | -0.1624 | Negative | Negative | Negative | 25.3333333 | Living |
| MB-5551 | 0.0459 | Negative | Negative | Negative | 182.233333 | Living |
| MB-5560 | -0.3812 | Negative | Negative | Negative | 186.6 | Living |
| MB-5566 | -0.3918 | Negative | Negative | Negative | 234.4 | Living |
| MB-5572 | -0.3023 | Negative | Negative | Negative | 178.633333 | Living |
| MB-5577 | -0.3075 | Negative | Negative | Negative | 55.4 | Died of Disease |
| MB-5602 | -0.243 | Negative | Negative | Negative | 224.433333 | Living |
| MB-5616 | -0.4177 | Negative | Negative | Negative | 180.633333 | Living |
| MB-5624 | -0.0001 | Negative | Negative | Negative | 136 | Living |
| MB-5633 | -0.2307 | Negative | Negative | Negative | 35.0333333 | Died of Disease |
| MB-5634 | -0.3129 | Negative | Negative | Negative | 62.9 | Died of Disease |
| MB-5651 | 0.0192 | Negative | Negative | Negative | 21 | Died of Disease |
| MB-5655 | -0.2177 | Negative | Negative | Negative | 191.8 | Living |
| MB-6052 | -0.2067 | Negative | Negative | Negative | 255.366667 | Living |
| MB-6055 | -0.3166 | Negative | Negative | Negative | 200.433333 | Living |
| MB-6058 | -0.1607 | Negative | Negative | Negative | 22.6666667 | Died of Disease |
| MB-6062 | -0.209 | Negative | Negative | Negative | 282.366667 | Living |
| MB-6098 | -0.1614 | Negative | Negative | Negative | 10.6333333 | Died of Disease |
| MB-6143 | -0.3368 | Negative | Negative | Negative | 255.6 | Living |

| | | | | | | |
|---|---|---|---|---|---|---|
| MB-6144 | -0.2779 | Negative | Negative | Negative | 254.933333 | Living |
| MB-6152 | -0.6098 | Negative | Negative | Negative | 228.9 | Living |
| MB-6178 | -0.1453 | Negative | Negative | Negative | 33.1333333 | Died of Disease |
| MB-6188 | -0.4752 | Negative | Negative | Negative | 23.7666667 | Living |
| MB-6223 | -0.3411 | Negative | Negative | Negative | 221.933333 | Living |
| MB-6242 | -0.5495 | Negative | Negative | Negative | 165.433333 | Living |
| MB-6245 | -0.1497 | Negative | Negative | Negative | 177.633333 | Living |
| MB-6248 | 0.3035 | Negative | Negative | Negative | 182.833333 | Living |
| MB-6251 | 0.049 | Negative | Negative | Negative | 14.7 | Died of Disease |
| MB-6272 | 0.0155 | Negative | Negative | Negative | 22.4 | Died of Disease |
| MB-6280 | -0.1243 | Negative | Negative | Negative | 27.8666667 | Died of Disease |
| MB-6305 | -0.2051 | Negative | Negative | Negative | 89.5333333 | Died of Disease |
| MB-6336 | 0.0358 | Negative | Negative | Negative | 17.2 | Died of Disease |
| MB-7007 | 0.1025 | Negative | Negative | Negative | 58.6 | Living |
| MB-7009 | -0.593 | Negative | Negative | Negative | 54.7666667 | Died of Disease |
| MB-7012 | -0.3488 | Negative | Negative | Negative | 19.8333333 | Died of Disease |
| MB-7017 | -0.2599 | Negative | Negative | Negative | 135.333333 | Living |
| MB-7023 | -0.5324 | Negative | Negative | Negative | 27.9666667 | Died of Disease |
| MB-7025 | 0.2476 | Negative | Negative | Negative | 80.2333333 | Living |
| MB-7031 | -0.6269 | Negative | Negative | Negative | 85.3666667 | Living |
| MB-7036 | -0.3656 | Negative | Negative | Negative | 120.433333 | Living |
| MB-7038 | 0.0432 | Negative | Negative | Negative | 81.0333333 | Living |
| MB-7039 | -0.0881 | Negative | Negative | Negative | 49.2 | Died of Disease |
| MB-7045 | -0.336 | Negative | Negative | Negative | 94.9333333 | Died of Disease |
| MB-7049 | -0.246 | Negative | Negative | Negative | 102.033333 | Living |
| MB-7052 | -0.5739 | Negative | Negative | Negative | 106.133333 | Living |
| MB-7055 | -0.1814 | Negative | Negative | Negative | 9.13333333 | Died of Disease |
| MB-7057 | -0.2173 | Negative | Negative | Negative | 43.8333333 | Died of Disease |
| MB-7066 | -0.3547 | Negative | Negative | Negative | 89.7666667 | Living |
| MB-7078 | 0.032 | Negative | Negative | Negative | 4.16666667 | Died of Disease |
| MB-7081 | -0.4424 | Negative | Negative | Negative | 140.233333 | Living |
| MB-7089 | -0.116 | Negative | Negative | Negative | 118.9 | Living |
| MB-7090 | -0.2107 | Negative | Negative | Negative | 109.766667 | Living |
| MB-7114 | -0.4688 | Negative | Negative | Negative | 123.266667 | Living |
| MB-7119 | -0.1304 | Negative | Negative | Negative | 128.366667 | Living |
| MB-7145 | -0.1078 | Negative | Negative | Negative | 138.333333 | Living |
| MB-7151 | -0.1756 | Negative | Negative | Negative | 144.433333 | Living |
| MB-7155 | -0.4125 | Negative | Negative | Negative | 149.766667 | Living |
| MB-7158 | -0.0698 | Negative | Negative | Negative | 46.4333333 | Died of Disease |
| MB-7159 | -0.3751 | Negative | Negative | Negative | 21.9333333 | Died of Disease |
| MB-7165 | -0.1426 | Negative | Negative | Negative | 147.166667 | Living |
| MB-7205 | -0.2726 | Negative | Negative | Negative | 166.033333 | Living |

| | | | | | | |
|---|---|---|---|---|---|---|
| MB-7208 | -0.1829 | Negative | Negative | Negative | 45.9333333 | Died of Disease |
| MB-7225 | -0.1587 | Negative | Negative | Negative | 55 | Died of Disease |
| MB-7258 | -0.2877 | Negative | Negative | Negative | 15.0666667 | Died of Disease |
| MB-7269 | -0.2828 | Negative | Negative | Negative | 21.1666667 | Died of Disease |
| MB-7270 | -0.4258 | Negative | Negative | Negative | 175.166667 | Living |

## Source Data for Figure 2A&B

2A: The densitometric analysis of the α9 nAChR overexpression efficiencies in HCC38 (n=3).

| Ctrl | | | CHRNA9 | | |
|---|---|---|---|---|---|
| 1 | 1 | 1 | 1.79649883 | 1.6584027 | 1.79878063 |

2A: The densitometric analysis of the α9 nAChR knockdown efficiencies in HCC38 (n=3).

| shCtrl | | | shCHRNA9-1 | | | shCHRNA9-2 | | |
|---|---|---|---|---|---|---|---|---|
| 1 | 1 | 1 | 0.48251462 | 0.58609773 | 0.34591611 | 0.37042674 | 0.54526493 | 0.29036228 |

2B: The densitometric analysis of the α9 nAChR overexpression efficiencies in HCC1937 (n=3).

| Ctrl | | | CHRNA9 | | |
|---|---|---|---|---|---|
| 1 | 1 | 1 | 2.61712222 | 1.77315655 | 1.79498122 |

2B: The densitometric analysis of the α9 nAChR knockdown efficiencies in HCC1937 (n=3).

| shCtrl | | | shCHRNA9-1 | | | shCHRNA9-2 | | |
|---|---|---|---|---|---|---|---|---|
| 1 | 1 | 1 | 0.5992965 | 0.57391399 | 0.58660525 | 0.31709639 | 0.33162066 | 0.32435853 |

## Source Data for Figure 2C-F

2C: The cell growth analysis of the α9 nAChR overexpression effect in HCC38 examined by ATP assay (n=3).

| | Ctrl | | | CHRNA9 | | |
|---|---|---|---|---|---|---|
| 0h | 100 | 100 | 100 | 100 | 100 | 100 |
| 24h | 267.7632 | 272.7503 | 268.5289 | 296.8131 | 290.2479 | 293.7451 |
| 48h | 284.2597 | 263.9055 | 279.4072 | 336.7444 | 359.7435 | 347.5384 |
| 72h | 335.5258 | 324.1066 | 328.7619 | 436.1879 | 426.2505 | 421.8799 |
| 96h | 379.1963 | 356.3882 | 365.7184 | 542.0764 | 521.7569 | 552.5762 |

2D: The cell growth analysis of the α9 nAChR knockdown effect in HCC38 examined by ATP assay (n=3).

| | shCtrl | | | shCHRNA9-1 | | | shCHRNA9-2 | | |
|---|---|---|---|---|---|---|---|---|---|
| 0h | 100 | 100 | 100 | 100 | 100 | 100 | 100 | 100 | 100 |
| 24h | 127.0767 | 118.3735 | 132.9748 | 103.7316 | 110.0027 | 99.9185 | 116.0848 | 105.7303 | 111.1445 |
| 48h | 159.0405 | 143.9795 | 153.0785 | 121.136 | 132.8194 | 122.7298 | 128.6243 | 124.9025 | 130.6737 |
| 72h | 214.0803 | 202.4384 | 213.3996 | 179.6485 | 196.776 | 195.9059 | 132.7674 | 134.6125 | 152.6572 |
| 96h | 314.2592 | 349.2464 | 383.7378 | 216.3415 | 237.7063 | 230.1026 | 165.7281 | 190.7541 | 193.3195 |

2E: The cell growth analysis of the α9 nAChR overexpression effect in HCC1937 examined by ATP assay (n=3).

| | Ctrl | | | CHRNA9 | | |
|---|---|---|---|---|---|---|
| 0h | 100 | 100 | 100 | 100 | 100 | 100 |
| 24h | 142.21544 | 129.071995 | 133.359873 | 150.510109 | 129.05224 | 143.080436 |
| 48h | 234.162169 | 231.651348 | 229.482869 | 248.574807 | 218.589227 | 220.013861 |
| 72h | 336.345015 | 316.764834 | 322.08892 | 367.084001 | 304.489992 | 314.923691 |
| 96h | 395.803676 | 382.943313 | 374.329233 | 445.748932 | 432.371334 | 457.343009 |

2F: The cell growth analysis of the α9 nAChR knockdown effect in HCC1937 examined by ATP assay (n=3).

| | shCtrl | | | shCHRNA9-1 | | | shCHRNA9-2 | | |
|---|---|---|---|---|---|---|---|---|---|
| 0h | 100 | 100 | 100 | 100 | 100 | 100 | 100 | 100 | 100 |
| 24h | 121.776 | 108.5989 | 107.9851 | 100.7073 | 119.3902 | 123.3846 | 117.4057 | 123.6551 | 125.4649 |
| 48h | 173.9171 | 161.7151 | 164.909 | 144.5265 | 147.0089 | 165.7876 | 155.2232 | 153.054 | 151.5182 |
| 72h | 218.3406 | 190.8942 | 193.348 | 187.7191 | 194.7245 | 185.6744 | 183.4288 | 176.0905 | 166.1094 |
| 96h | 220.7747 | 201.9488 | 208.4937 | 178.5603 | 200.8667 | 192.6365 | 168.9521 | 171.2382 | 189.2431 |

## Source Data for Figure 2G-J

2G: The cell growth analysis of the α9 nAChR overexpression effect in HCC38 examined by colony formation experiment (n=3).

| Ctrl | | | CHRNA9 | | |
|---|---|---|---|---|---|
| 12 | 15 | 9 | 45 | 47 | 54 |

2H: The cell growth analysis of the α9 nAChR overexpression effect in HCC1937 examined by colony formation experiment (n=3).

| Ctrl | | | CHRNA9 | | |
|---|---|---|---|---|---|
| 3 | 3 | 5 | 15 | 20 | 29 |

2I: The cell growth analysis of the α9 nAChR knockdown effect in HCC38 examined by colony formation experiment (n=3).

| shCtrl | | | shCHRNA9-1 | | | shCHRNA9-2 | | |
|---|---|---|---|---|---|---|---|---|
| 43 | 49 | 47 | 25 | 22 | 22 | 4 | 5 | 8 |

2J: The cell growth analysis of the α9 nAChR knockdown effect in HCC1937 examined by colony formation experiment (n=3).

| shCtrl | | | shCHRNA9-1 | | | shCHRNA9-2 | | |
|---|---|---|---|---|---|---|---|---|
| 44 | 75 | 64 | 6 | 5 | 9 | 5 | 6 | 5 |

## Source Data for Figure 3B-I, K-R

3B: The densitometric analysis of the α9 nAChR overexpression effect on phospho-AKT (Ser473) /AKT ratio in HCC38 (n=3).

| Ctrl | | | CHRNA9 | | |
|---|---|---|---|---|---|
| 1 | 1 | 1 | 1.66043194 | 1.73722665 | 1.6988293 |

3C: The densitometric analysis of the α9 nAChR overexpression effect on phospho-ERK (Thr202/Tyr204) /ERK ratio in HCC38 (n=3).

| Ctrl | | | CHRNA9 | | |
|---|---|---|---|---|---|
| 1 | 1 | 1 | 2.17546288 | 2.4564343 | 2.31594859 |

3D: The densitometric analysis of the α9 nAChR overexpression effect on phospho-STAT3 (Tyr705) /STAT3 ratio in HCC38 (n=3).

| Ctrl | | | CHRNA9 | | |
|---|---|---|---|---|---|
| 1 | 1 | 1 | 2.36429172 | 2.3542387 | 2.35926521 |

3E: The densitometric analysis of the α9 nAChR overexpression effect on BAX/Bcl-2 ratio in HCC38 (n=3).

| Ctrl | | | CHRNA9 | | |
|---|---|---|---|---|---|
| 1 | 1 | 1 | 0.83339903 | 1.02768562 | 0.92661028 |

3F: The densitometric analysis of the α9 nAChR knockdown effect on phospho-AKT (Ser473) /AKT ratio in HCC38 (n=3).

| shCtrl | | | shCHRNA9-1 | | | shCHRNA9-2 | | |
|---|---|---|---|---|---|---|---|---|
| 1 | 1 | 1 | 0.59604555 | 0.36918971 | 0.48261763 | 0.58749471 | 0.52301827 | 0.55525649 |

3G: The densitometric analysis of the α9 nAChR knockdown effect on phospho-ERK (Thr202/Tyr204) /ERK ratio in HCC38 (n=3).

| shCtrl | | | shCHRNA9-1 | | | shCHRNA9-2 | | |
|---|---|---|---|---|---|---|---|---|
| 1 | 1 | 1 | 0.60399989 | 0.5696626 | 0.58683125 | 0.60399989 | 0.5696626 | 0.58683125 |

3H: The densitometric analysis of the α9 nAChR knockdown effect on phospho-STAT3 (Tyr705) /STAT3 ratio in HCC38 (n=3).

| shCtrl | | | shCHRNA9-1 | | | shCHRNA9-2 | | |
|---|---|---|---|---|---|---|---|---|
| 1 | 1 | 1 | 0.5697224 | 0.3979573 | 0.48383985 | 0.59298473 | 0.45889363 | 0.52593918 |

3I: The densitometric analysis of the α9 nAChR knockdown effect on BAX/Bcl-2 ratio in HCC38 (n=3).

| shCtrl | | | shCHRNA9-1 | | | shCHRNA9-2 | | |
|---|---|---|---|---|---|---|---|---|
| 1 | 1 | 1 | 0.94637174 | 1.47633078 | 1.09735388 | 0.96390974 | 1.33924135 | 1.08400518 |

3K: The densitometric analysis of the α9 nAChR overexpression effect on phospho-AKT (Ser473) /AKT ratio in HCC1937 (n=3).

| Ctrl | | | CHRNA9 | | |
|---|---|---|---|---|---|
| 1 | 1 | 1 | 1.61576034 | 1.62314758 | 1.61945396 |

3L: The densitometric analysis of the α9 nAChR overexpression effect on phospho-ERK (Thr202/Tyr204) /ERK ratio in HCC1937 (n=3).

| Ctrl | | | CHRNA9 | | |
|---|---|---|---|---|---|
| 1 | 1 | 1 | 1.56693221 | 1.52397924 | 1.54545573 |

3M: The densitometric analysis of the α9 nAChR overexpression effect on phospho-STAT3 (Tyr705) /STAT3 ratio in HCC1937 (n=3).

| Ctrl | | | CHRNA9 | | |
|---|---|---|---|---|---|
| 1 | 1 | 1 | 1.83049743 | 1.86739784 | 1.84894764 |

3N: The densitometric analysis of the α9 nAChR overexpression effect on BAX/Bcl-2 ratio in HCC1937 (n=3).

| Ctrl | | | CHRNA9 | | |
|---|---|---|---|---|---|
| 1 | 1 | 1 | 1.27646281 | 1.06560163 | 1.17103222 |

3O: The densitometric analysis of the α9 nAChR knockdown effect on phospho-AKT (Ser473) /AKT ratio in HCC1937 (n=3).

| shCtrl | | | shCHRNA9-1 | | | shCHRNA9-2 | | |
|---|---|---|---|---|---|---|---|---|
| 1 | 1 | 1 | 0.55520106 | 0.50208146 | 0.52864126 | 0.48586536 | 0.46259605 | 0.47423071 |

3P: The densitometric analysis of the α9 nAChR knockdown effect on phospho-ERK (Thr202/Tyr204) /ERK ratio in HCC1937 (n=3).

| shCtrl | | | shCHRNA9-1 | | | shCHRNA9-2 | | |
|---|---|---|---|---|---|---|---|---|
| 1 | 1 | 1 | 0.75128571 | 0.81405387 | 0.78266979 | 0.75128571 | 0.81405387 | 0.78266979 |

3Q: The densitometric analysis of the α9 nAChR knockdown effect on phospho-STAT3 (Tyr705) /STAT3 ratio in HCC1937 (n=3).

| shCtrl | | | shCHRNA9-1 | | | shCHRNA9-2 | | |
|---|---|---|---|---|---|---|---|---|
| 1 | 1 | 1 | 0.57140103 | 0.36303454 | 0.46721779 | 0.58952542 | 0.50338095 | 0.54645319 |

3R: The densitometric analysis of the α9 nAChR knockdown effect on BAX/Bcl-2 ratio in HCC1937 (n=3).

| shCtrl | | | shCHRNA9-1 | | | shCHRNA9-2 | | |
|---|---|---|---|---|---|---|---|---|
| 1 | 1 | 1 | 0.79010084 | 1.05142592 | 0.92076338 | 0.74073958 | 0.77098315 | 0.75586137 |

## Source Data for Figure 4A-L

4A: The quantitative analysis of the α9 nAChR overexpression effect on intracellular ROS levels in HCC38 (n=3).

| Ctrl | | | CHRNA9 | | |
|---|---|---|---|---|---|
| 10528 | 9989 | 10157 | 8877 | 9198 | 8718 |

4B: The quantitative analysis of the α9 nAChR knockdown effect on intracellular ROS levels in HCC38 (n=3).

| shCtrl | | | shCHRNA9-1 | | | shCHRNA9-2 | | |
|---|---|---|---|---|---|---|---|---|
| 1976 | 1977 | 1930 | 2554 | 2514 | 2508 | 2561 | 2521 | 2444 |

4C: The quantitative analysis of the α9 nAChR overexpression effect on intracellular ROS levels in HCC1937 (n=3).

| Ctrl | | | CHRNA9 | | |
|---|---|---|---|---|---|
| 1211 | 1160 | 1148 | 777 | 789 | 746 |

4D: The quantitative analysis of the α9 nAChR knockdown effect on intracellular ROS levels in HCC1937 (n=3).

| shCtrl | | | shCHRNA9-1 | | | shCHRNA9-2 | | |
|---|---|---|---|---|---|---|---|---|
| 486 | 478 | 467 | 561 | 555 | 551 | 462 | 481 | 448 |

4E: The quantitative analysis of the α9 nAChR overexpression effect on MDA levels in HCC38 (n=3).

| Ctrl | | | CHRNA9 | | |
|---|---|---|---|---|---|
| 2.222807 | 2.680199 | 2.083488 | 1.679443 | 1.833826 | 1.869907 |

4F: The quantitative analysis of the α9 nAChR knockdown effect on MDA levels in HCC38 (n=3).

| shCtrl | | | shCHRNA9-1 | | | shCHRNA9-2 | | |
|---|---|---|---|---|---|---|---|---|
| 0.3131 | 0.283 | 0.3052 | 0.4722 | 0.4604 | 0.4575 | 0.4425 | 0.4327 | 0.4454 |

4G: The quantitative analysis of the α9 nAChR overexpression effect on MDA levels in HCC1937 (n=3).

| Ctrl | | | CHRNA9 | | |
|---|---|---|---|---|---|
| 11.07122 | 11.42349 | 10.62941 | 3.016262 | 2.224238 | 5.199517 |

4H: The quantitative analysis of the α9 nAChR knockdown effect on MDA levels in HCC1937 (n=3).

| shCtrl | | | shCHRNA9-1 | | | shCHRNA9-2 | | |
|---|---|---|---|---|---|---|---|---|
| 0.3183 | 0.3111 | 0.3168 | 0.3726 | 0.383 | 0.3675 | 0.43 | 0.4253 | 0.4336 |

4I: The quantitative analysis of the α9 nAChR overexpression effect on GSH levels in HCC38 (n=3).

| Ctrl | | | CHRNA9 | | |
|---|---|---|---|---|---|
| 3.258961 | 3.091375 | 2.738113 | 6.707712 | 6.573357 | 6.91003 |

4J: The quantitative analysis of the α9 nAChR knockdown effect on GSH levels in HCC38 (n=3).

| shCtrl | | | shCHRNA9-1 | | | shCHRNA9-2 | | |
|---|---|---|---|---|---|---|---|---|
| 4.860587 | 4.780286 | 5.02644 | 3.552195 | 3.965545 | 3.226517 | 2.307635 | 2.372027 | 1.932851 |

4K: The quantitative analysis of the α9 nAChR overexpression effect on GSH levels in HCC1937 (n=3).

| Ctrl | | | CHRNA9 | | |
|---|---|---|---|---|---|
| 2.805655 | 2.860922 | 2.839333 | 4.746023 | 5.573651 | 5.314396 |

4L: The quantitative analysis of the α9 nAChR knockdown effect on GSH levels in HCC1937 (n=3).

| shCtrl | | | shCHRNA9-1 | | | shCHRNA9-2 | | |
|---|---|---|---|---|---|---|---|---|
| 5.862765 | 6.155279 | 6.290656 | 5.270956 | 5.615274 | 5.758939 | 5.057274 | 5.114683 | 5.17238 |

## Source Data for Figure 5C-L

5C: The densitometric analysis of the α9 nAChR overexpression effect on SLC7A11 in HCC38 (n=3).

| Ctrl | | | CHRNA9 | | |
|---|---|---|---|---|---|
| 1 | 1 | 1 | 1.46268374 | 1.78267666 | 1.89712439 |

5D: The densitometric analysis of the α9 nAChR overexpression effect on GPX4 in HCC38 (n=3).

| Ctrl | | | CHRNA9 | | |
|---|---|---|---|---|---|
| 1 | 1 | 1 | 2.57581263 | 2.47409637 | 2.26383002 |

5E: The densitometric analysis of the α9 nAChR overexpression effect on Keap1 in HCC38 (n=3).

| Ctrl | | | CHRNA9 | | |
|---|---|---|---|---|---|
| 1 | 1 | 1 | 0.4976386 | 0.47924521 | 0.48844191 |

5F: The densitometric analysis of the α9 nAChR overexpression effect on Nrf2 in HCC38 (n=3).

| Ctrl | | | CHRNA9 | | |
|---|---|---|---|---|---|
| 1 | 1 | 1 | 1.67559089 | 1.95278562 | 1.81418826 |

5G: The densitometric analysis of the α9 nAChR overexpression effect on HO-1 in HCC38 (n=3).

| Ctrl | | | CHRNA9 | | |
|---|---|---|---|---|---|
| 1 | 1 | 1 | 1.6573638 | 2.05992489 | 1.6573638 |

5H: The densitometric analysis of the α9 nAChR knockdown effect on SLC7A11 in HCC38 (n=3).

| shCtrl | | | shCHRNA9-1 | | | shCHRNA9-2 | | |
|---|---|---|---|---|---|---|---|---|
| 1 | 1 | 1 | 0.53304506 | 0.52652272 | 0.52978389 | 0.51554828 | 0.52652272 | 0.5210355 |

5I: The densitometric analysis of the α9 nAChR knockdown effect on GPX4 in HCC38 (n=3).

| shCtrl | | | shCHRNA9-1 | | | shCHRNA9-2 | | |
|---|---|---|---|---|---|---|---|---|
| 1 | 1 | 1 | 0.6609848 | 0.51518725 | 0.51518725 | 0.64097724 | 0.47734576 | 0.47734576 |

5J: The densitometric analysis of the α9 nAChR knockdown effect on Keap1 in HCC38 (n=3).

| shCtrl | | | shCHRNA9-1 | | | shCHRNA9-2 | | |
|---|---|---|---|---|---|---|---|---|
| 1 | 1 | 1 | 1.60200123 | 1.33259498 | 1.26369115 | 1.40481444 | 2.1918762 | 2.2971307 |

5K The densitometric analysis of the α9 nAChR knockdown effect on Nrf2 in HCC38 (n=3).

| shCtrl | | | shCHRNA9-1 | | | shCHRNA9-2 | | |
|---|---|---|---|---|---|---|---|---|
| 1 | 1 | 1 | 0.66138421 | 0.61137009 | 0.63637715 | 0.70009717 | 0.59234561 | 0.64622139 |

5L: The densitometric analysis of the α9 nAChR overexpression effect on HO-1 in HCC38 (n=3).

| shCtrl | | | shCHRNA9-1 | | | shCHRNA9-2 | | |
|---|---|---|---|---|---|---|---|---|
| 1 | 1 | 1 | 0.57627719 | 0.60752941 | 0.50843295 | 0.55684588 | 0.5867832 | 0.38897344 |

## Source Data for Figure 6B

6B: The endpoint tumor weight of HCC38 xenograft nude mice to evaluate anti-tumor effect of α9 nAChR knockdown.

| shCtrl | shCHRNA9 |
|---|---|
| 155.2 mg | 32 mg |
| 227.6 mg | 7.3 mg |
| 126.9 mg | 28.6 mg |
| 59 mg | 18.9 mg |
| 67.4 mg | 13.6 mg |
| 38.5 mg | 11.4 mg |

## Source Data for Figure 6E

6E: The endpoint tumor weight of 4T1 allograft mice to evaluate anti-tumor effect of α9 nAChR antagonist GeXIVA[1,2].

| Vehicle | GeXIVA |
|---|---|
| 239.02 mg | 142.75 mg |
| 211.02 mg | 110.96 mg |
| 163.05 mg | 98.06 mg |
| 162.74 mg | 97.66 mg |
| 156.25 mg | 39.54 mg |
| 123.56 mg | 20.36 mg |

# Source Data for Western Blot Images

## Figure 1

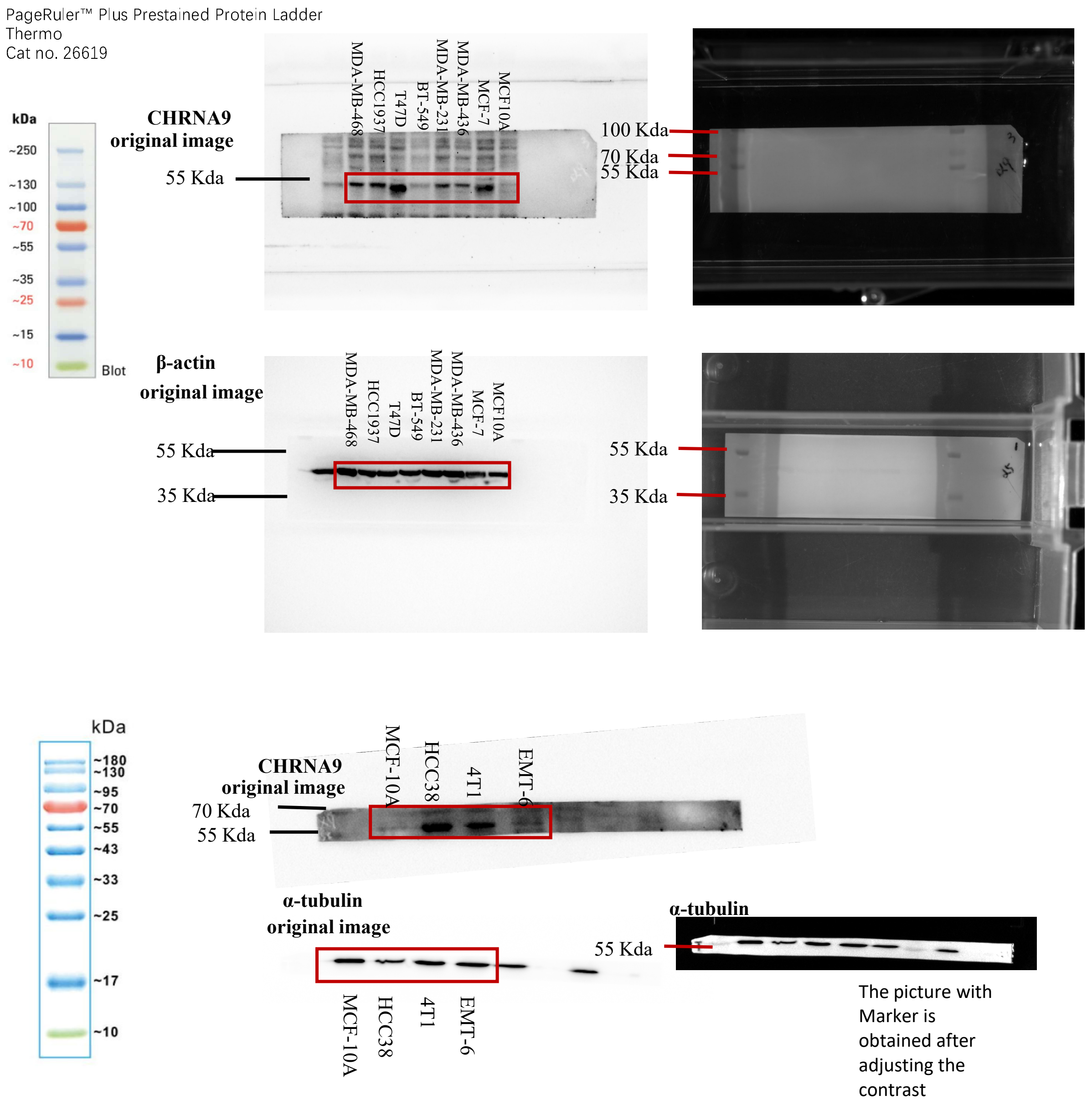

# Figure 2

α9 nAChR

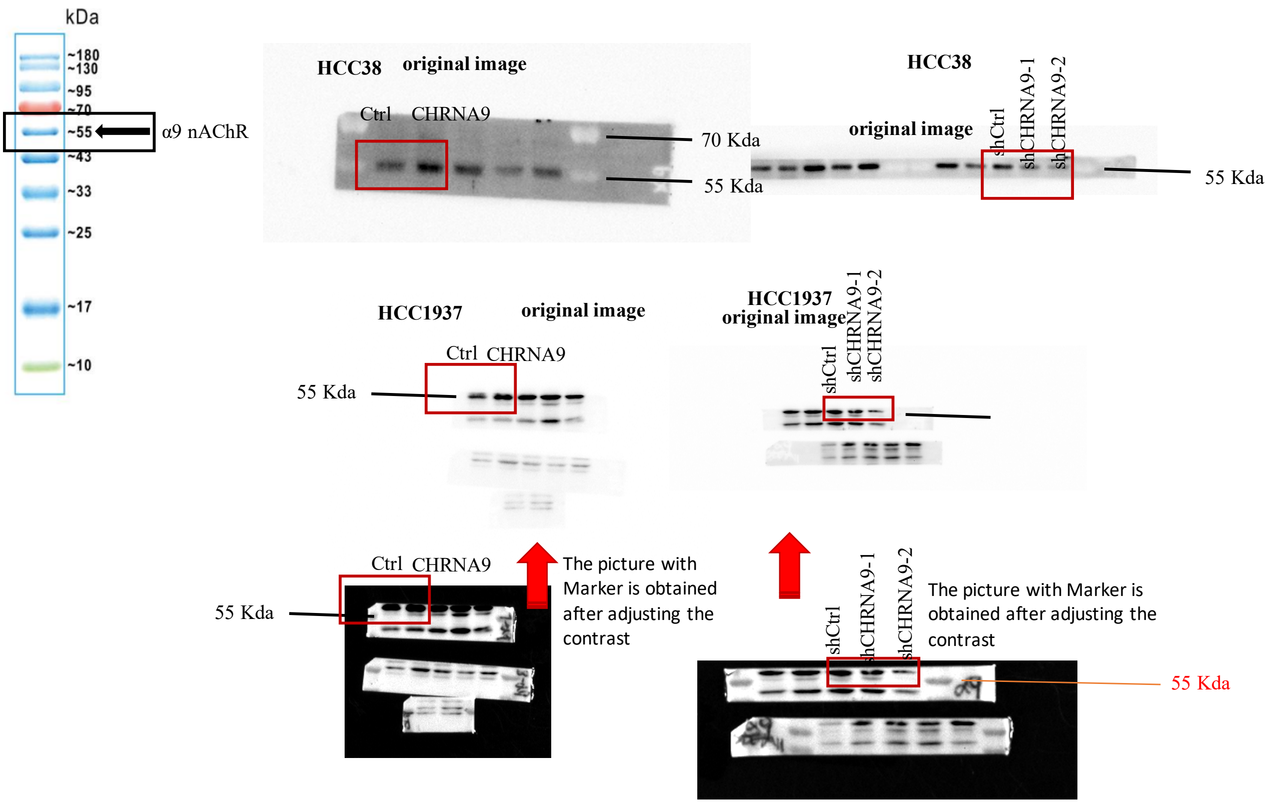


GAPDH

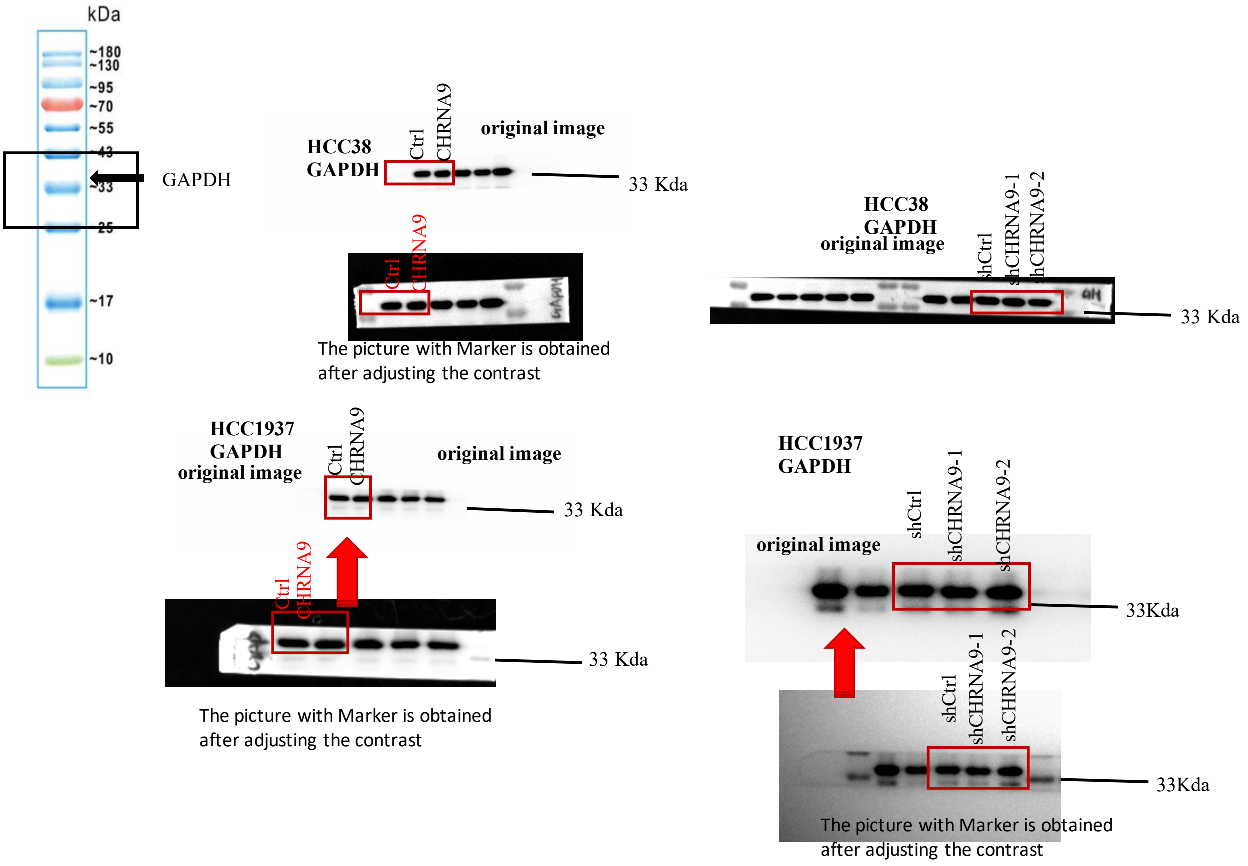

# Figure 3

## p-AKT

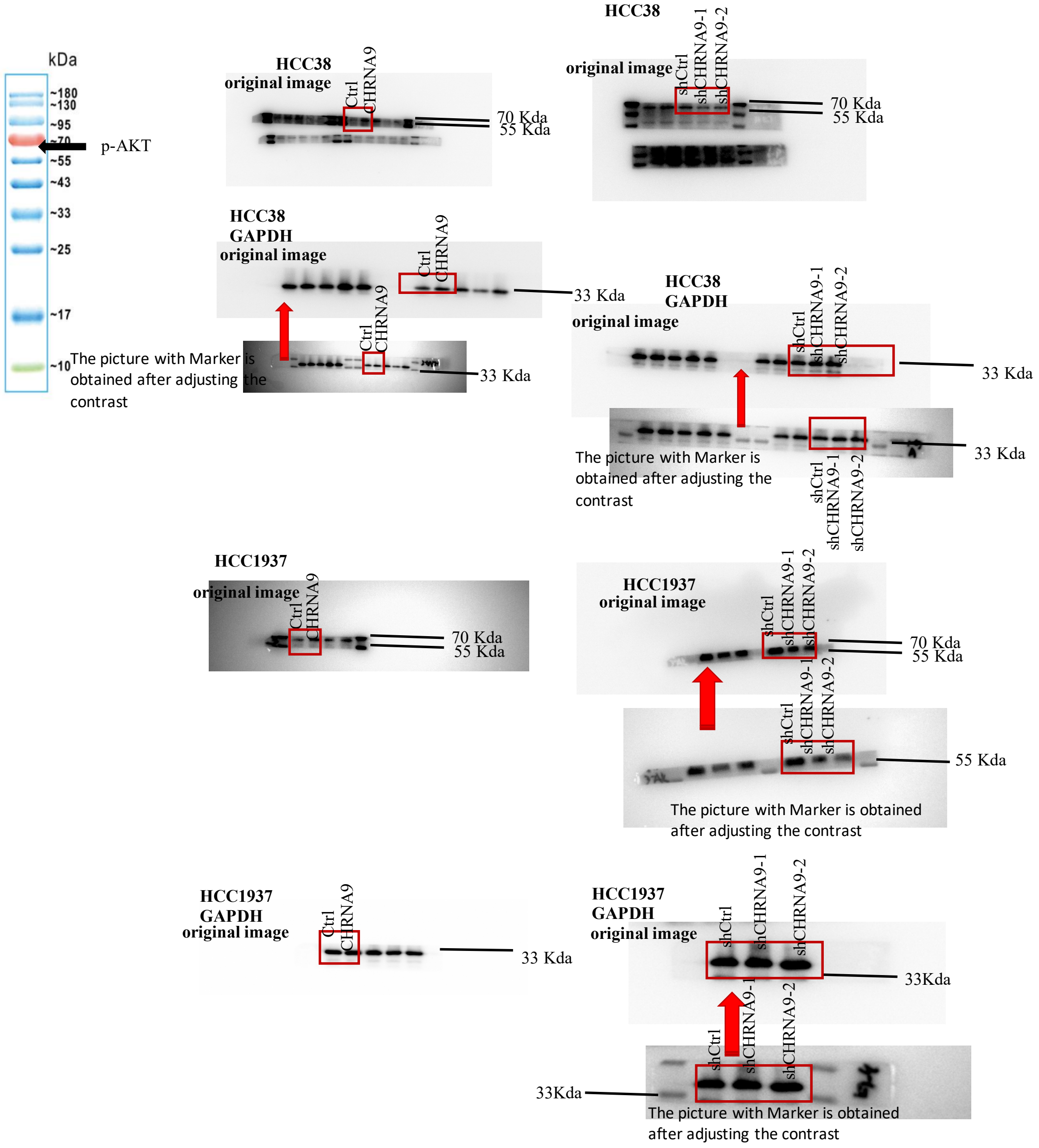


## AKT

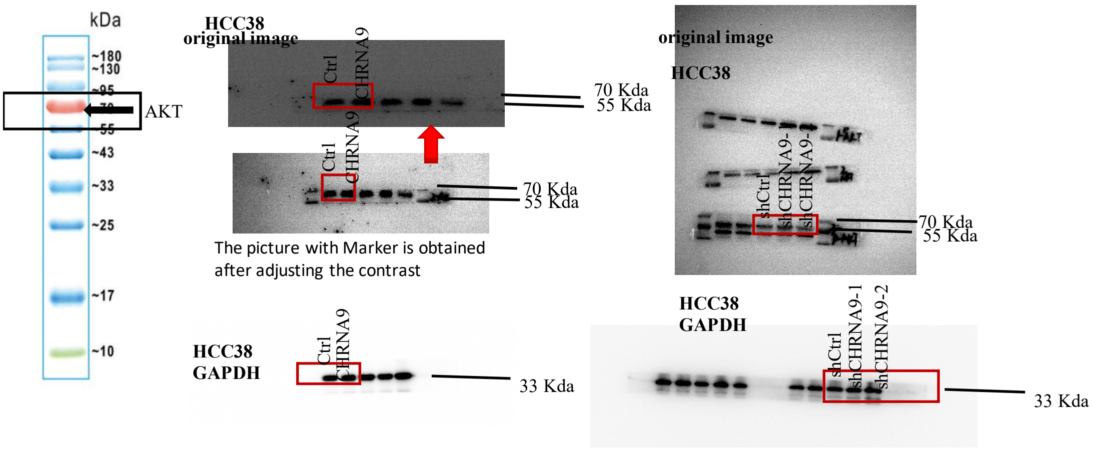

# Figure 3 (continued)

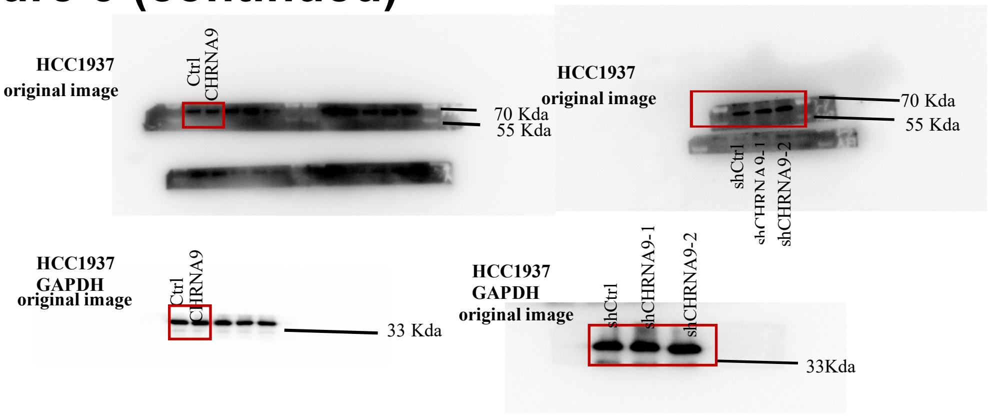


p-ERK

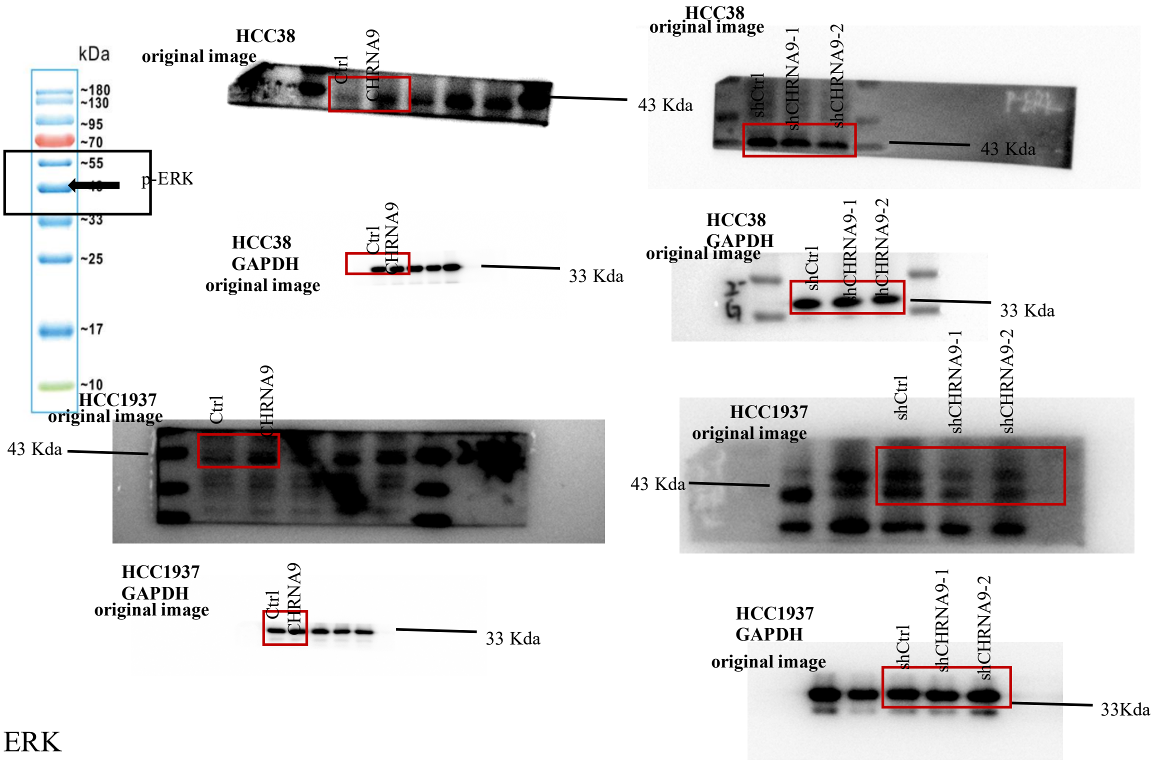


ERK

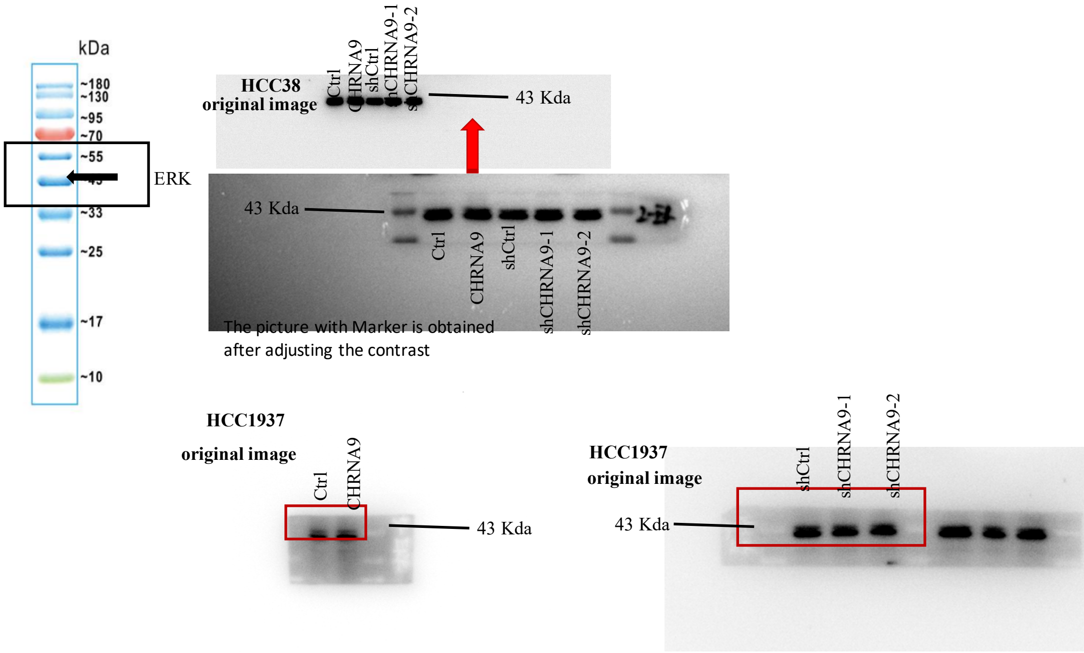

# Figure 3 (continued)

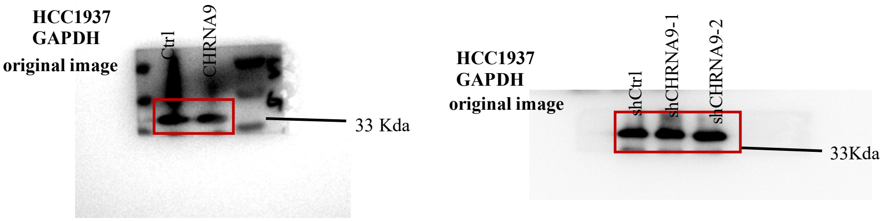


p-STAT3

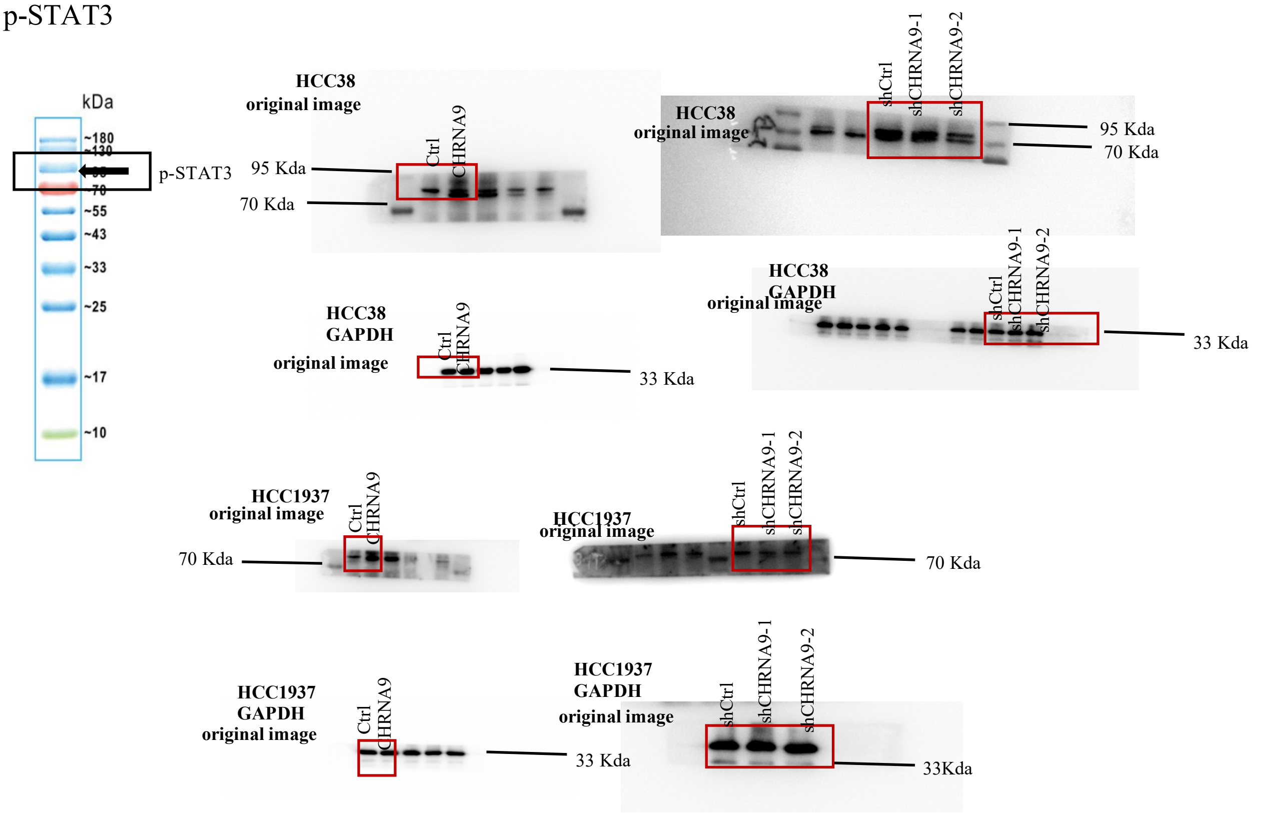


STAT3

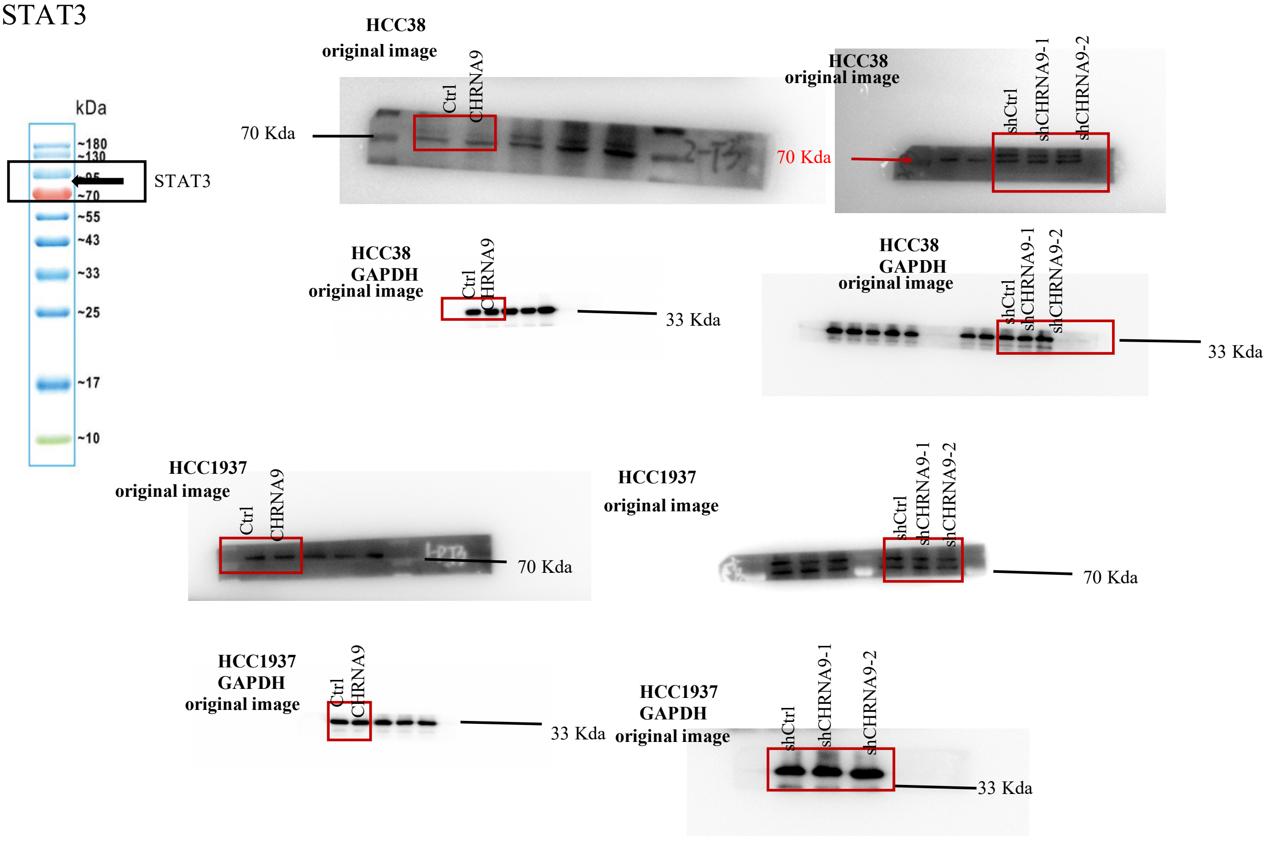

# Figure 3 (continued)

Bcl-2

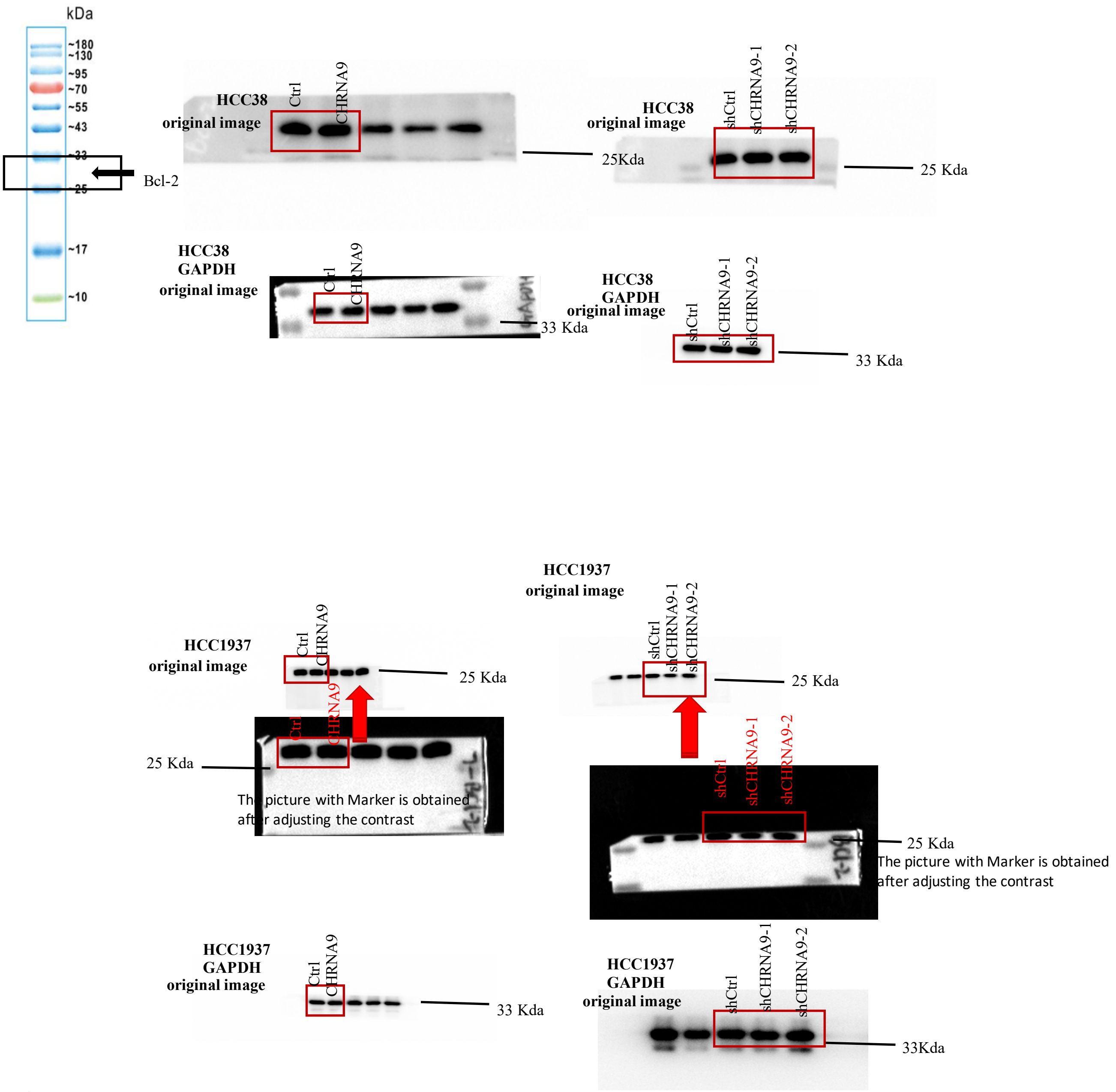


BAX

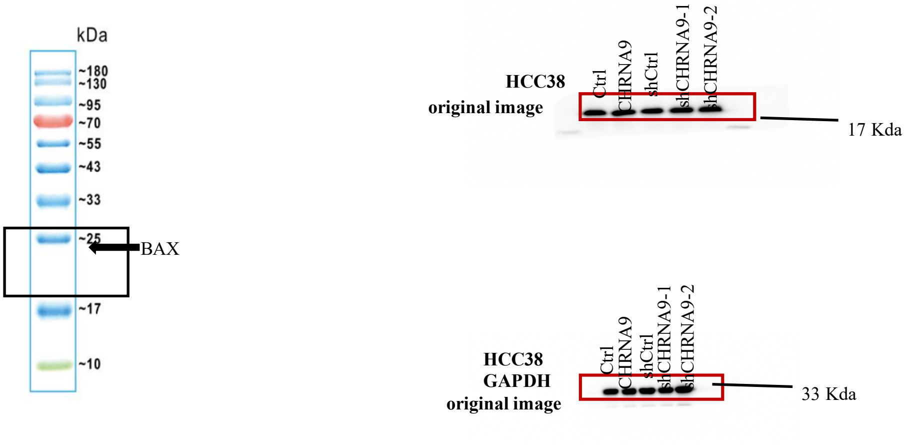

# Figure 3 (continued)

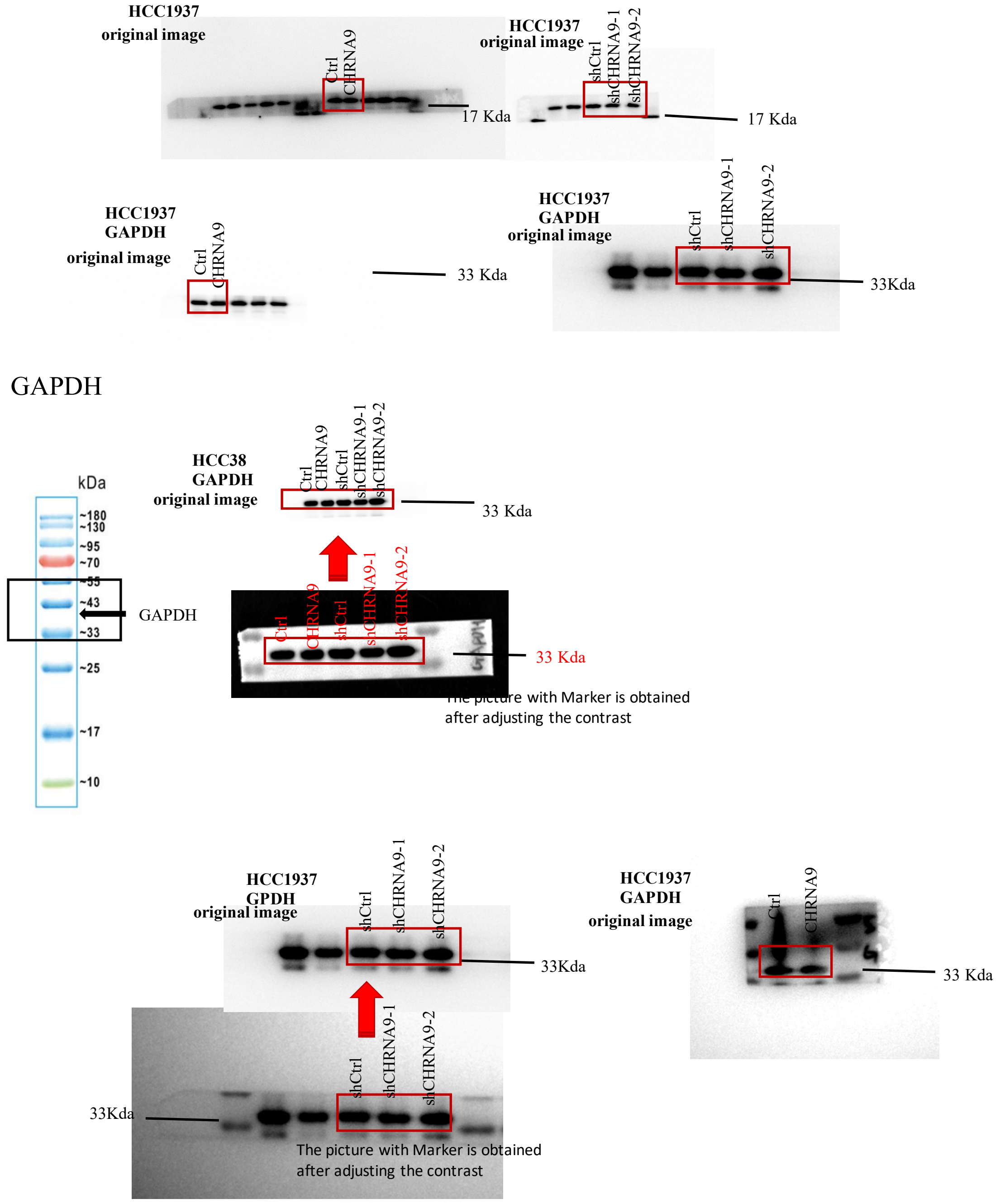

# Figure 5

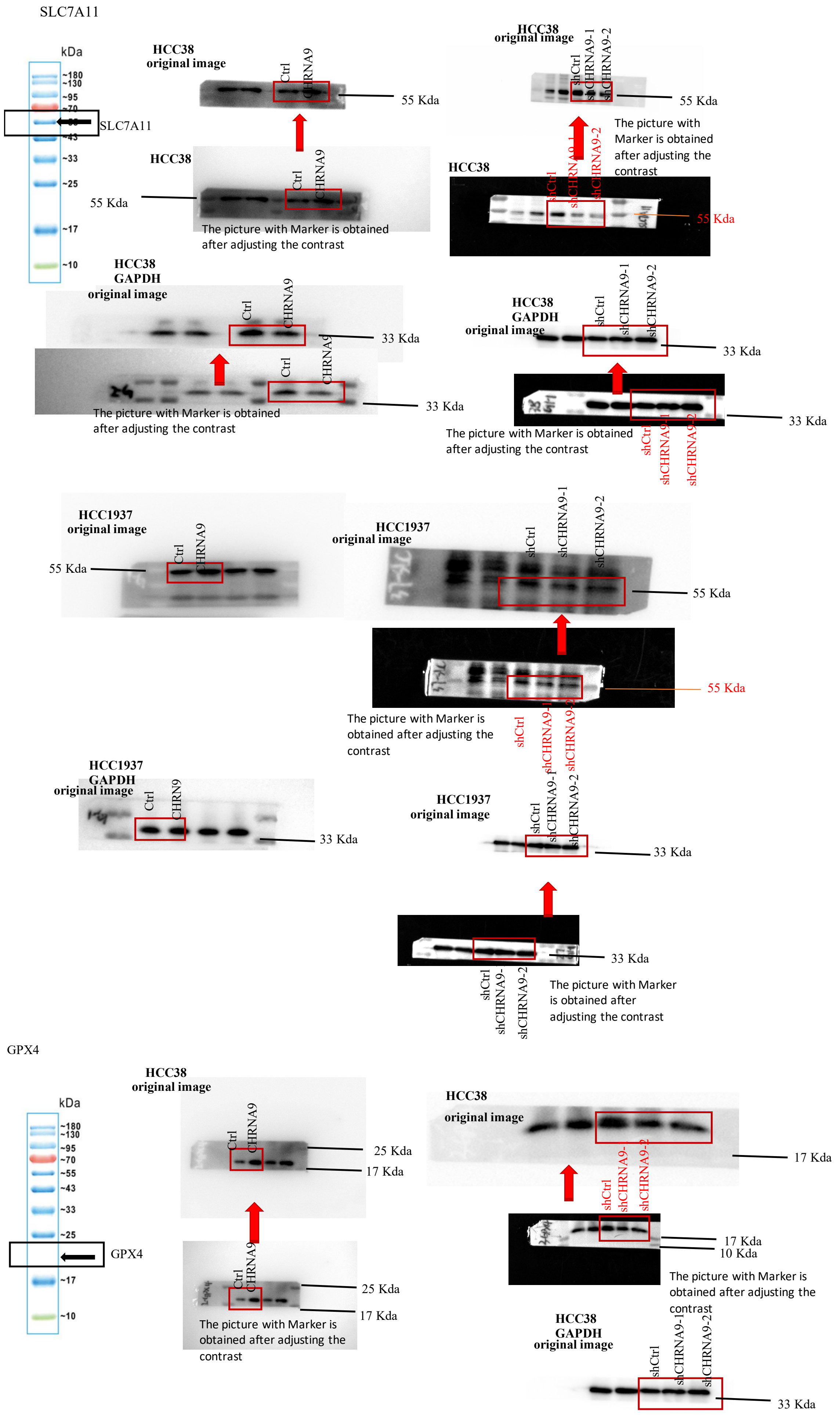
SLC7A11
kDa
~180
~130
~95
~70
~43
~33
~25
~17
~10
SLC7A11
HCC38
original image
Ctrl
CHRNA9
55 Kda
HCC38
55 Kda
The picture with Marker is obtained after adjusting the contrast
HCC38
original image
shCtrl
shCHRNA9-1
shCHRNA9-2
55 Kda
The picture with Marker is obtained after adjusting the contrast
HCC38
shCtrl
shCHRNA9-2
55 Kda
HCC38
GAPDH
original image
Ctrl
CHRNA9
33 Kda
The picture with Marker is obtained after adjusting the contrast
33 Kda
HCC38
GAPDH
original image
shCtrl
shCHRNA9-1
shCHRNA9-2
33 Kda
The picture with Marker is obtained after adjusting the contrast
33 Kda
shCtrl
shCHRNA9-1
shCHRNA9-2
HCC1937
original image
55 Kda
Ctrl
CHRNA9
HCC1937
original image
shCtrl
shCHRNA9-1
shCHRNA9-2
55 Kda
55 Kda
The picture with Marker is obtained after adjusting the contrast
shCtrl
shCHRNA9-1
shCHRNA9-2
HCC1937
GAPDH
original image
Ctrl
CHRN9
33 Kda
HCC1937
original image
shCtrl
shCHRNA9-1
shCHRNA9-2
33 Kda
33 Kda
shCtrl
shCHRNA9-
shCHRNA9-2
The picture with Marker is obtained after adjusting the contrast
GPX4
kDa
~180
~130
~95
~70
~55
~43
~33
~25
~17
~10
GPX4
HCC38
original image
Ctrl
CHRNA9
25 Kda
17 Kda
25 Kda
17 Kda
The picture with Marker is obtained after adjusting the contrast
HCC38
original image
17 Kda
shCtrl
shCHRNA9-
shCHRNA9-2
17 Kda
10 Kda
The picture with Marker is obtained after adjusting the contrast
HCC38
GAPDH
original image
shCtrl
shCHRNA9-1
shCHRNA9-2
33 Kda

# Figure 5 (continued)

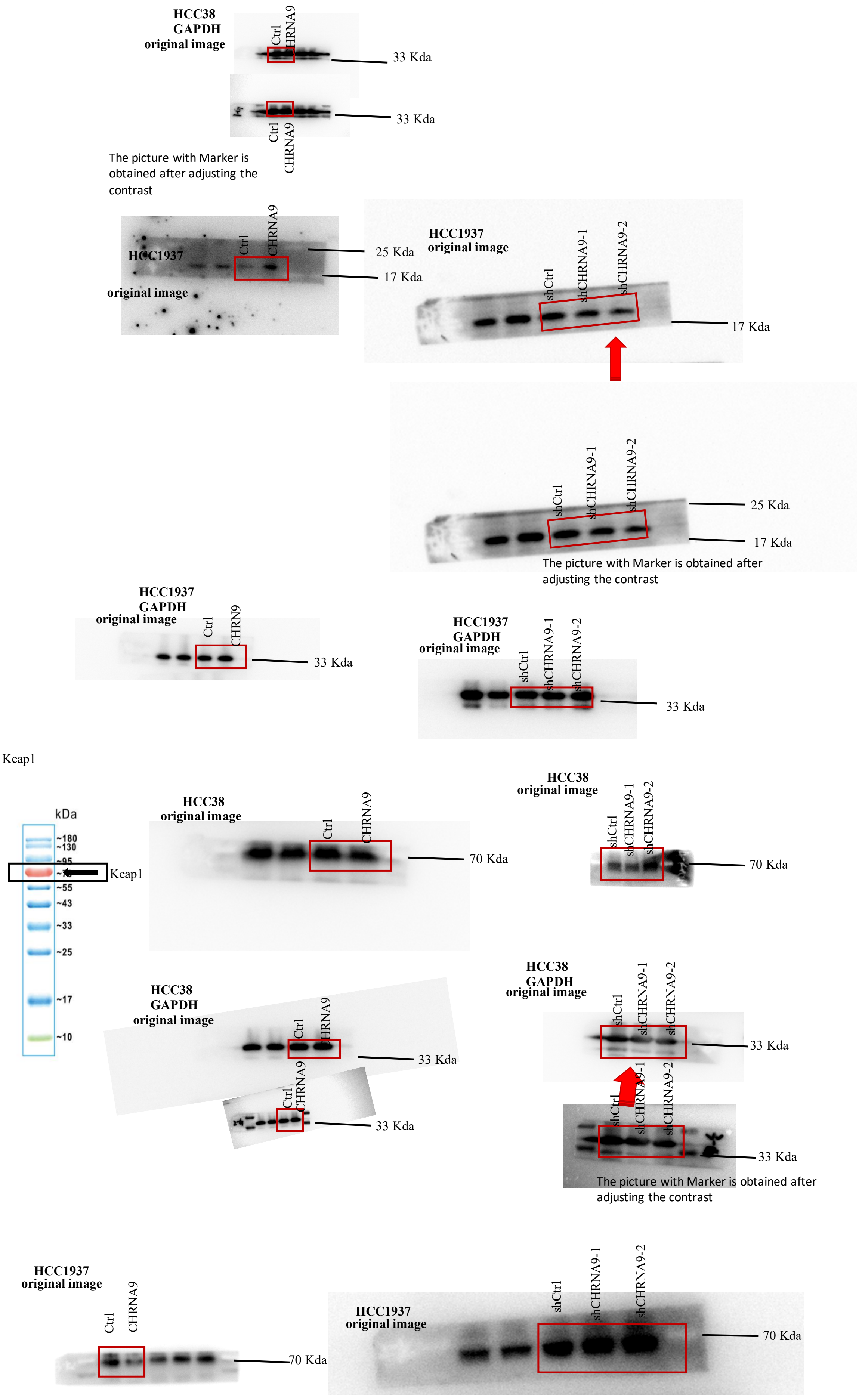

# Figure 5 (continued)

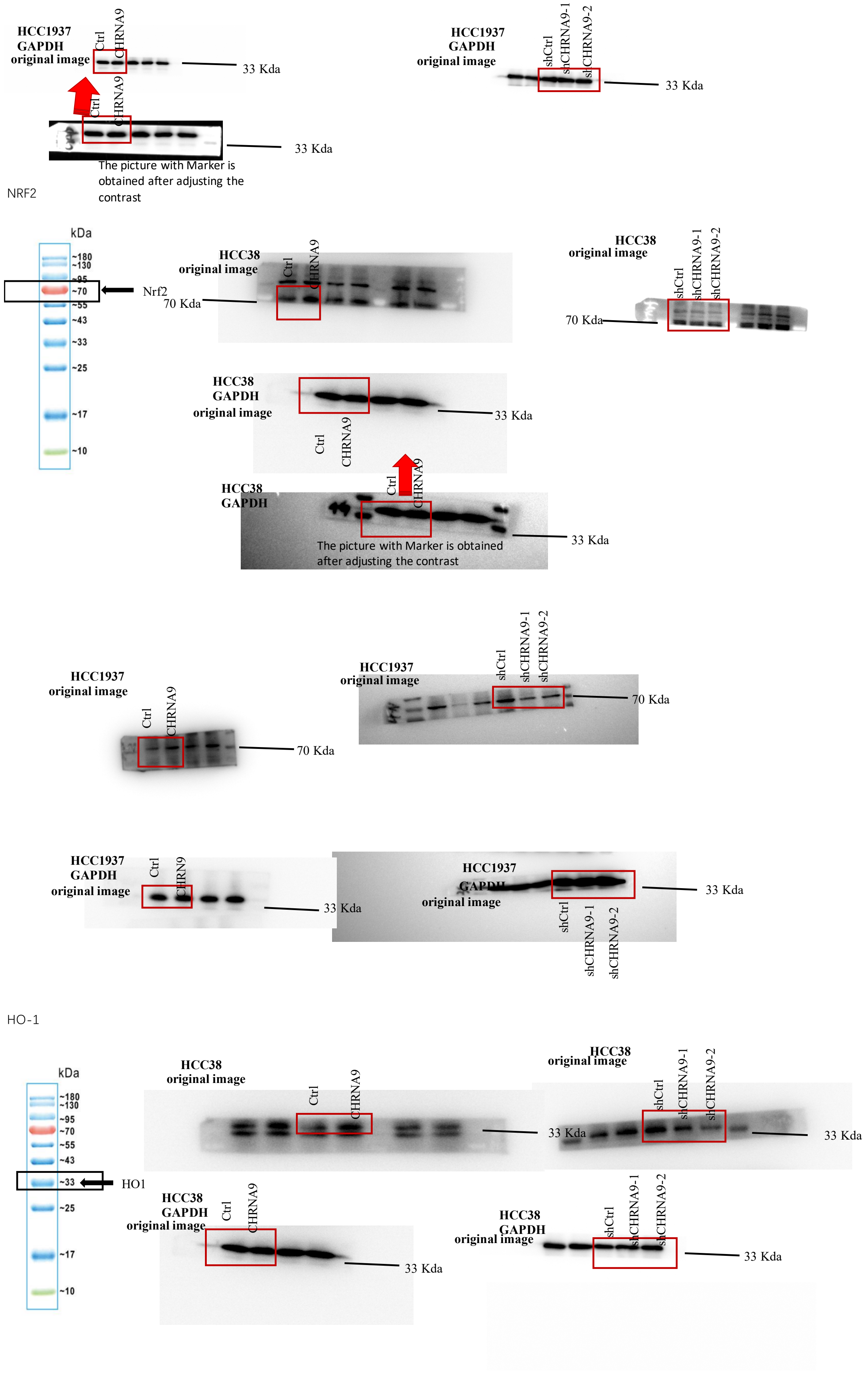

# Figure 5 (continued)

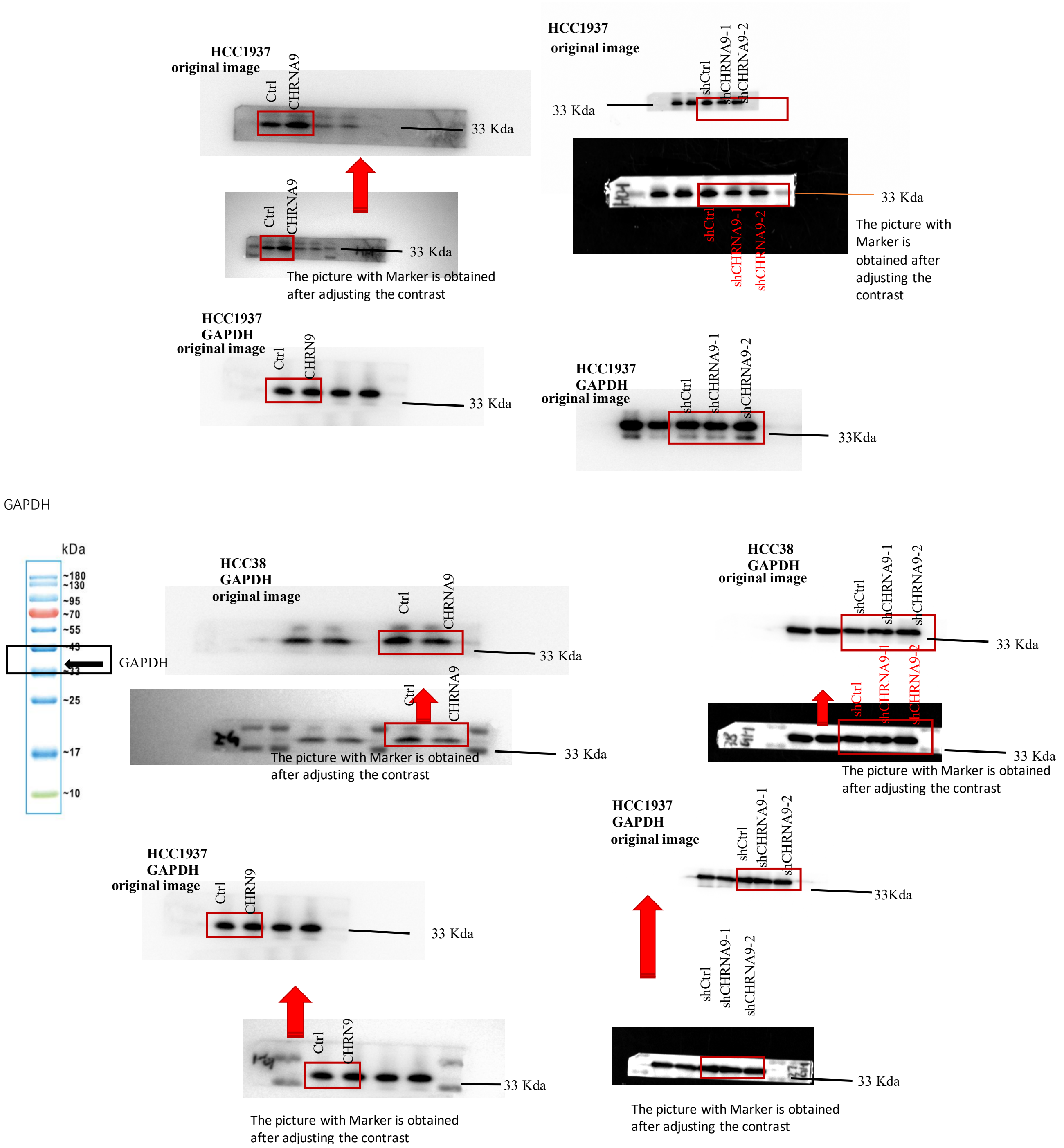

Supplement: Supplementary file 1 [file biomolecules-15-00835-s001.zip › biomolecules-3538472-supplementary.pdf]
